# Efficient reduction of nitro compounds and domino preparation of 1-substituted-1*H*-1,2,3,4-tetrazoles by Pd(ii)-polysalophen coated magnetite NPs as a robust versatile nanocomposite†

**DOI:** 10.1039/d1ra01164b

**Published:** 2021-03-30

**Authors:** DaPeng Xu, Meilu Xiong, Milad Kazemnejadi

**Affiliations:** Institute of Chemical Technology, Gansu Industry Polytechnic College TianShui 741000 China; Public Foundation College, Gansu Health Vocational College Lanzhou 730207 China bearbabyx@sina.com; Department of Chemistry, Faculty of Sciences, University of Birjand Birjand Iran miladkazemnejad@yahoo.com

## Abstract

A new, versatile, and green methodology has been developed for the efficient NaBH_4_-reduction of nitroarenes as well as the domino/reduction MCR preparation of 1-substituted-1*H*-1,2,3,4-tetrazoles using Pd(ii)-polysalophen coated magnetite NPs as an efficient heterogeneous magnetically recyclable nanocatalyst. Polysalophen was firstly prepared based on a triazine framework with a high degree of polymerization, then coordinated to Pd ions and, finally, the resulting hybrid was immobilized on magnetite NPs. The catalyst was characterized by various instrumental and analytical methods, including GPC, DLS, N_2_ adsorption–desorption, TGA, VSM, TEM, HRTEM, EDX, XPS, XRD, and ICP analyses. The catalyst possesses dual-functionality including the reduction of nitroarenes and the construction of tetrazole rings all in one step *via* a domino protocol. High to excellent yields were obtained for both nitro reduction and the direct preparation of 1-substituted-1*H*-1,2,3,4-tetrazoles from nitro compounds. Insight into the mechanism was conducted by XPS *in situ* as well as DLS *in situ* along with several control experiments. Recyclability of the catalyst was studied for 6 consecutive runs along with metal leaching measurements in each cycle.

## Abstract 2

Facile nitroarene reduction as well as domino/reduction MCR preparation of 1-substituted-1*H*-1,2,3,4-tetrazoles from nitroarenes was performed by Pd(ii)-polysalophen coated magnetite NPs as a highly selective, recyclable and efficient nanocomposite.

## Graphical abstract

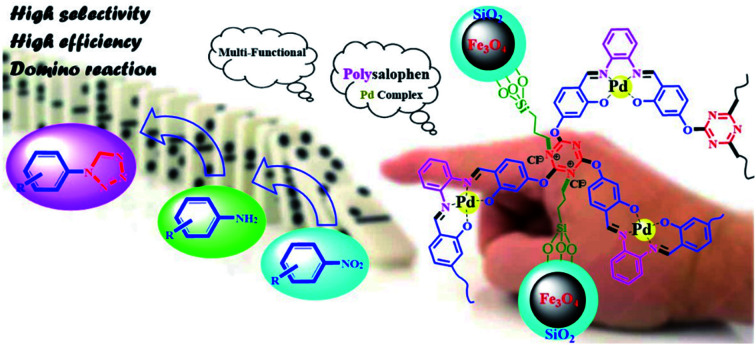

## Introduction

Amines and their derivatives are among the most practical raw materials and intermediates in organic synthesis that are widely used in the production of chemicals required in pharmaceuticals, biological compounds, dyes, synthetic resins, photography, agrochemicals, optical brighteners, rubber, and other industries.^1–3^ Moreover, the preparation of azo compounds, isocyanates, amides, imines, and diazonium salts, are some of the prominent uses of amines in organic synthesis.^4,5^ The complete reduction of nitro compounds to the corresponding amines is one of the most popular and common processes in the petrochemical, pharmaceutical, chemical and other industries.^1^ Various catalytic systems have been reported for the complete reduction of nitro compounds to amines, and recently we could point to: Ag-rGO/g-C_3_N_4_/visible light,^6^ human hair,^4^ Ni_2_BH_2_,^7^ Fe_3_O_4_-MWCNTs@PEI-Ag,^2^ Fe/TRGO,^1^ CuFe_2_O_4_/NaBH_4_,^8^ and Zn powder/CuSO_4_.^6^ Recently, Nasrollahzadeh *et al.* studied recent methodologies in graphene-based photocatalysts for nitro reduction in a review paper.^5^ Mahmoudi *et al.* recently developed a three-functional redox catalytic system bearing a TEMPO/Co(iii)-porphyrin/Ni(ii) complex for the efficient reduction of nitro compounds as well as its direct transformation to 1-substituted-1*H*-1,2,3,4-tetrazoles.^9^

Amines are one of the most valuable precursors in organic synthesis, especially MCRs (multicomponent reactions),^10^ and one of these reactions is the formation of tetrazole derivatives.

1-Substituted-1*H*-1,2,3,4-tetrazoles are one of the main groups of tetrazoles, and the most common method for their preparation is cyclization between a susceptible compound such as an amine in the presence of azide ions.^1,11–13^ Tetrazoles have prominent biological activities in pharmaceuticals and medicine, such as antifungal, antibacterial, anti-inflammatory, antihypertensive, antibiotic, anti-HIV, and anticonvulsant agents.^14–16^ Tetrazole rings can be found in Sartans as a large group of commercial pharmaceutical compounds.^10,17^ Moreover, different applications have been known for tetrazole derivatives in agriculture, photography, and various other industries.^10,17^ Various methodologies have been developed for the synthesis of 1-substituted-1*H*-1,2,3,4-tetrazoles,^10^ and we can point to heterogeneous catalysts, photocatalysts, microwave-assisted synthesis, nanoparticles, miscellaneous methods, *etc.*^10,12^

1-Substituted-1*H*-1,2,3,4-tetrazoles are usually made from amines. Direct preparation of these compounds from cheaper and more available precursors of nitro compounds is very valuable and noteworthy (compared to amines).^9^ However, the use of nitro compounds to prepare tetrazole compounds requires an additional step of the complete reduction of the nitro compounds to amines. So, designing and developing a multifunctional catalytic system for reduction and MCR/domino processes seems to be the smart/correct strategy.

Domino reactions, also known as cascade or tandem reactions, are chemical processes that involve at least two consecutive reactions, making it possible to prepare the desired products with simpler and more accessible precursors, and the reaction proceeds through intermediates, each of which alone can lead to the desired product. For example, Nasseri *et al.* reported domino protocols for the preparation of benzimidazole derivatives and Biginelli reactions from alcohols using a Cu–Mn bimetallic complex immobilized on magnetic NPs.^18^ Hydroformylation/aldol condensation,^19^ synthesis of cyclic amidines,^20^ one-pot synthesis of vinylsilanes,^21^ synthesis of polysubstituted pyrroles and lamellarin R,^22^ and synthesis of multisubstituted tetrahydrobenzofuran derivatives^23^ are some recent applications of domino reactions in organic synthesis.

**Scheme 1:**
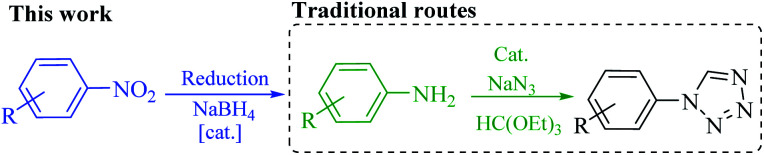
Direct preparation of 1-substituted-1*H*-1,2,3,4-tetrazole from nitro compounds in this work, and its comparison with the traditional approach.

**Scheme 2:**
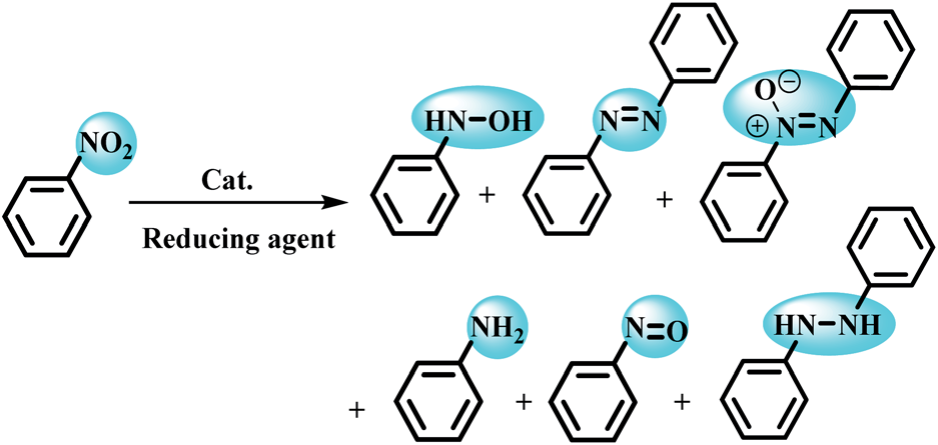
Six possible products from the reduction of nitrobenzene.

Despite the development of various catalytic systems in this field, the design and synthesis of a comprehensive catalyst with high catalytic activity and selectivity is the main concern of scientists in the field of synthesis of organic compounds *via* the domino protocol. From this viewpoint, the reduction of nitro compounds generally includes a multi-step reaction with the formation of various by-products. So, if the applied catalyst does not act selectively, it produces several by-products that are not favorable from work-up, product isolation, or cost viewpoints in either industry or organic synthesis. Scheme 1 shows the six possible products that could be formed during nitrobenzene reduction in the presence of a reducing agent. As shown in Scheme 2, the complete reduction of nitrobenzene gives aniline. In this way, catalyst selectivity along with its high efficiency are two inseparable characteristics for a catalyst to be applicable in the reduction processes.

Salen and its derivatives are known as among the most reliable and active ligands in coordination chemistry due to their rigid 4-dentate structure. The use of salen complexes has been extended to almost all organic reactions, especially coupling reactions and MCR reactions.^24^ The immobilization of these compounds on heterogeneous supports and their use as heterogeneous catalysts in organic synthesis has been an interesting topic for researchers over the last decade, because it increases the efficiency of the catalyst and reduces costs. The polymerization of these compounds is an intelligent strategy to achieve this goal. However, due to the structure of salen ligands, its polymerization has many challenges and few reports are available on the preparation of poly-salen and its derivatives. Previously, Yao *et al.*^25^ and Song *et al.*^26^ in two separate studies developed two novel chiral poly-salen Mn(iii) complexes derived from (*R*,*R*)-2-diaminocyclohexane and disalicylaldehydes for the enantioselective epoxidation of unfunctionalized olefins. Wang *et al.* reported a new approach for the preparation of poly-salen based on polyacrylamide and used its Pd complex for a carbonylative Sonogashira reaction.^27^ Wu and Lu synthesized a series of poly-salens through polycondensation of 1,2-diamine with a disalicylaldehyde for the dimerization of propylene.^28^ Recently, a Co(iii) salen polymer was prepared *via* a Sonogashira Pd cross-coupling reaction between tris(*p*-ethynyl)triphenylamine (TPA) and bis-bromo salen metal complexes.^29^

In this work, a polysalophen network structure was prepared through a polycondensation reaction and then immobilized on magnetite NPs. The polycondensation reaction was performed on a salicylaldehyde-treated triazine framework, where the polymerization occurs from three sides in the presence of *o*-phenylenediamine. Finally, the resulting polymer was successfully immobilized on magnetite NPs after the coordination of Pd ions to the polysalophen ligand. The resulting nanocomposite showed noteworthy catalytic activity towards the reduction of nitroarenes in the presence of NaBH_4_ and the domino/reduction multicomponent preparation of 1-substituted-1*H*-1,2,3,4-tetrazoles from nitroarenes (or aromatic amines) in one step.

## Experimental

### Materials and instrumentation

All materials were supplied by Merck (or Sigma) or the Fluka Corporation and used as received without any further purification. All solvents (except dimethyl sulfone) were dried before use and kept under an Ar atmosphere. The dimethyl sulfone used in the solvent-effect experiments was prepared according to the previously reported method described by Cheng *et al.*^30^ Molecular sieves with a 4 Å pore diameter, 0.6 (g mL^−1^) bulk density, 20% w/w adsorbed water, and 0.5 %w/w attrition were used for the polymerization as a water-absorbent agent and catalyst.

In optimization experiments, the conversion of each run was recorded on a YL 6100 gas chromatograph (GC) system with a CBP5 column (Shimadzu 30 m × 0.32 mm × 0.25 mm). Also, the conversion and selectivity of the products in the model reactions were determined qualitatively by GC analysis. Conversion was calculated by comparing the area of the product and the starting material. For each run, after catalyst recovery, 0.2 μL of a sample was injected into the GC instrument, then the selectivity of aniline production was obtained from the following eqn (1):^31^1
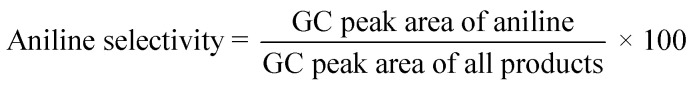


^1^H-NMR (400 MHz) and ^13^C-NMR (100 MHz) spectra were recorded on a Bruker Ascend NMR 400 MHz in ultra-pure deuterated solvents. TMS was used as an internal standard for all analyses. XPS analyses were performed on an XR3E2 (VG Microtech) twin anode X-ray source with radiation of Al-Kα = 1486.6 eV. TEM images were taken with a Philips EM208 microscope, operated at 100 kV. High resolution transmission electron microscopy (HRTEM) was performed on an FEI Tecnai G2 F20 Super Twin TEM microscope with a field emission gun at 200 kV. Zeta potentials and DLS (to study the size distribution of the samples) analyses of the samples were obtained using a zeta potential analyzer from Malvern Instruments Ltd. The thermal behavior of the samples was studied on a NETZSCH STA 409 PC/PG under N_2_ atmosphere with a heating rate of 10 °C min^−1^ in the temperature range of 25–850 °C. A Lake Shore vibrating sample magnetometer (VSM) was used for studying the magnetic properties of the samples. Metal leaching and Pd contents of the catalyst measurements were performed on a VARIAN VISTA-PRO CCD simultaneous ICP-OES instrument. A gel permeation chromatography Knauer Advanced Scientific Instrument (Germany) with an RI detector (Smartline 2300) PL gel 10 mm, 10 × 10^3^ Å column with an injected volume of 20 μL was used for the determination of the average molecular weight of the polysalophen. Monodispersed poly(methyl methacrylate) (PMMA) was used as a standard for calibration. The average molar weight was measured using Millennium 2010 software. Energy dispersive X-ray spectroscopy (EDX) analyses were accomplished on a field emission scanning electron microscope, FE-SEM, JEOL 7600F, equipped with an energy dispersive X-ray spectrometer from Oxford Instruments. The crystal structures of the samples were performed using a Bruker D8/Advance powder X-ray diffractometer, and using a HAAKE D8 recirculating bath the cell temperature was maintained at 25.0 °C. Elemental analyses of the tetrazole products were obtained on a CHN ThermoFinnigan Flash EA 1112 instrument. The specific surface area, pore diameter, and pore volume, of the obtained NPs were studied by N_2_ physisorption at −196 °C on a Micromeritics ASAP 2000 instrument by the BET method.

### Synthesis of 4,4′,4''-((1,3,5-triazine-2,4,6-triyl)tris(oxy))tris(2-hydroxybenzaldehyde) (3)

**Scheme 3:**
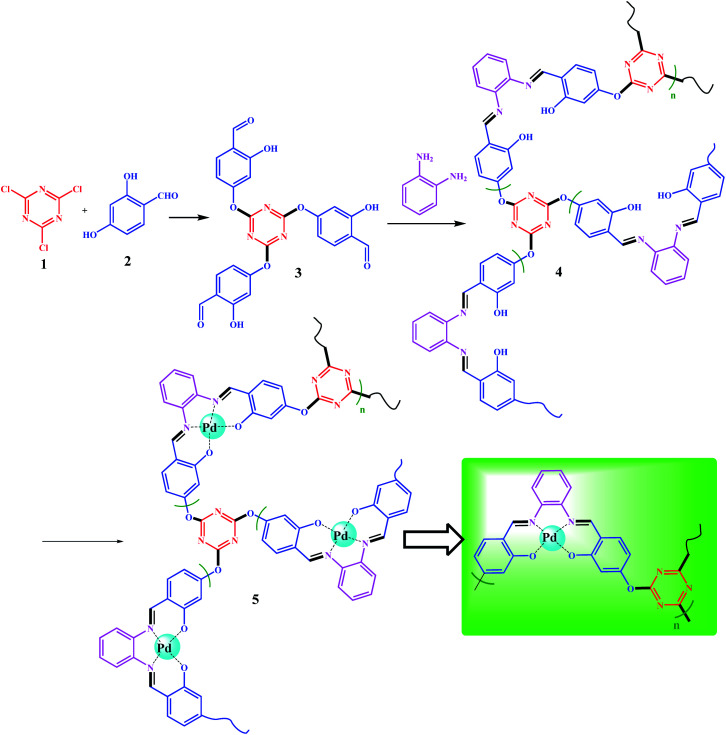
Preparation of polysalophen 4 and Pd(ii)-polysalophen complex 5.

4,4′,4′′-((1,3,5-Triazine-2,4,6-triyl)tris(oxy))tris(2-hydroxybenzaldehyde) abbreviated to TA(SA)_3_3, was prepared according to the following procedure: In a dried round bottom flask, cyanuric chloride (TCT, 1.0 mmol) was dissolved in 25 mL of dry CH_3_CN at room temperature. The system was equipped with an N_2_ inlet after the addition of 2,4-dihydroxybezaldehyde (5.0 mmol) and Et_3_N (5.0 mmol); these excess amounts were used to ensure that all chloride sites in TCT were functionalized by 2,4-dihydroxybezaldehyde. The reaction was performed under reflux conditions for 12 h. The dark yellow solid was filtered and washed with cold EtOH. Further purification was achieved by recrystallization (twice) in hot ethanol (to eliminate any possible mono- and di-substituted products) (Scheme 3). The product was isolated at 4 °C (0.73 mmol, 73% isolated yield, MP 145 °C). The structure of TA(SA)_3_3 was confirmed by CHN, ^1^H-NMR, ^13^C-NMR, and EDX analyses. Elemental analysis: calcd C: 58.90, H: 3.09, N: 8.59%, found. C: 58.76, H: 3.22, N: 8.60%. EDX analysis (wt%): C: 59.99, H: 3.09, N: 9.62%, O: 30.39. ^1^H NMR (400 MHz, CDCl_3_) *δ*(ppm) = 6.71 (s, 3H), 7.15 (d, 3H), 7.93 (d, 3H), 9.98 (s, 3H), 11.61 (s, 3H); ^13^C NMR (100 MHz, CDCl_3_) *δ*(ppm) = 109.4, 114.9, 118.7, 132.3, 162.3, 181.9, 195.4.

### Synthesis of polysalophen (4) based on TA(SA)_3_ polycondensation reaction

A polycondensation reaction on TA(SA)_3_ (3) was performed in the presence of *o*-phenylenediamine (*o*-PDA) based on salophen preparation. Dissolved TA(SA)_3_ (2.5 g, 5.0 mmol) in 30 mL of absolute EtOH was added dropwise to a ethanolic solution of *o*-PDA (1.6 g, 15.0 mmol in 35 mL of absolute EtOH) over 1.0 h. This excess amount was used to avoid dimerization as well as the formation of short chains. Seven beads of molecular sieves were added to the mixture. A molecular sieve not only catalyzes the reaction for the preparation of Schiff base bonds, but also absorbs the water produced during the polymerization process and enhances the polymerization reaction.^32^

### Synthesis of Pd(ii)-polysalophen complex 5

In order to prepare Pd(ii)-polysalophen complex 5, the polysalophen ligand (4) (1.0 g) was swelled in 25 mL of DMSO, then 0.01 g of Pd(OAc)_2_ (anhydrous) was added to the mixture. The mixture was stirred for 2.0 h at 80 °C. The resulting Pd(ii)-polysalophen complex was separated by simple filtration (centrifuges can also be used), followed by several washings with cold EtOH and deionized water. Finally, it was dried in an oven (50 °C) and isolated at room temperature.

### Synthesis of Fe_3_O_4_@SiO_2_@Pd(ii)-polysalophen 9

**Scheme 4:**
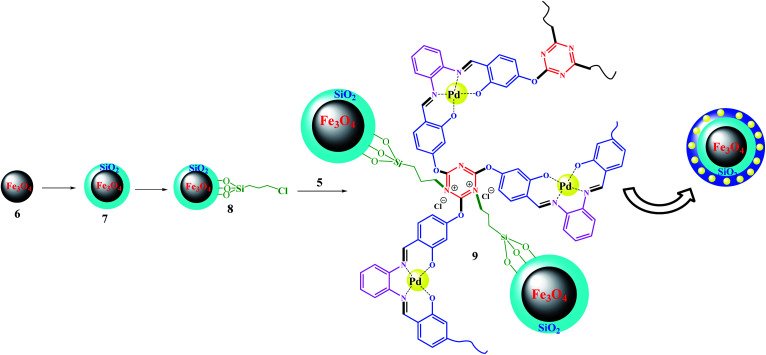
Preparation of Fe_3_O_4_@SiO_2_@polysalophen-Pd(ii) 9.

Fe_3_O_4_@SiO_2_–Cl was prepared according to a previously reported procedure.^33,34^ The immobilization process was accomplished by the following procedure: Fe_3_O_4_@SiO_2_–Cl was added to 15 mL of H_2_O : EtOH mixture (1 : 3, v/v), then sonicated for 15 min at 40 °C. In contrast, Pd(ii)-polysalophen was also sonicated in the same mixture for 15 min at 40 °C, then added slowly to the magnetite mixture under ultrasonic conditions. Next, the mixture was stirred for 4 h under reflux conditions. The resultant Fe_3_O_4_@SiO_2_@Pd(ii)-polysalophen was collected by an external magnetic field, washed with deionized water and dried in a vacuum oven for 8 h (Scheme 4).

### Catalytic complete reduction of nitroarenes to the corresponding amines catalyzed by Fe_3_O_4_@SiO_2_@Pd(ii)-polysalophen 9

In a typical run, in a reaction tube, nitrobenzene (1.0 mmol), NaBH_4_ (2.0 mmol), and catalyst 9 (5 mg, 0.3 mol% Pd) were added to 2.0 mL of H_2_O. The reaction was performed under reflux conditions while being monitored by TLC. Initial confirmation of product formation was done by comparing pure amine spots by TLC. Upon completion of the reaction, the catalyst was filtered magnetically, and the desired amine product was transferred to the organic phase after solvent extraction using EtOAc (2 × 15 mL). The organic phase was dried over MgSO_4_, then the solvent was removed under reduced pressure. Finally, the crude product was purified by flash chromatography. The products were confirmed by their CHN elemental analysis and MP and comparison with authentic references.

### General procedure for direct transformation of nitroarenes to 1-substituted-1*H*-1,2,3,4-tetrazoles catalyzed by Fe_3_O_4_@SiO_2_@Pd(ii)-polysalophen 9

In a reaction tube, nitro compound (1.0 mmol), NaBH_4_ (2.0 mmol), and catalyst 9 (5 mg, 0.3 mol% Pd), were added to 2.0 mL of H_2_O. The reaction was performed under reflux conditions and monitored by TLC. After 30 min of the mixture reacting, HC(OC_2_H_5_)_3_ (1.2 mmol) and NaN_3_ (1.5 mmol) were added to the mixture. The mixture was stirred for an appropriate time under reflux conditions. After completion of the reaction, the catalyst was filtered magnetically, washed with EtOH, dried and stored for the next run. Then, acetone (12 mL) was added to the residue, and the product was extracted with EtOAc (2 × 15 mL). The solvent was removed under reduced pressure and the desired crude product (1-substituted-1*H*-1,2,3,4-tetrazole) was purified by recrystallization. The products were characterized by ^1^H NMR, ^13^C NMR and compared with authentic ones.

### General procedure for transformation of amines to 1-substituted-1*H*-1,2,3,4-tetrazoles catalyzed by Fe_3_O_4_@SiO_2_@Pd(ii)-polysalophen 9

Aryl amine (1.0 mmol), HC(OC_2_H_5_)_3_ (1.2 mmol), catalyst 9 (5 mg, 0.3 mol% Pd), and NaN_3_ (1.5 mmol) were added to 2.0 mL of H_2_O. The mixture was stirred under reflux conditions for a sufficient time, and monitored by TLC according to amine consumption. Upon completion of the reaction, the mixture was cooled to room temperature, then extracted with EtOAc (3 × 15 mL) after removal of the catalyst by an external magnetic field. The organic phases were dried over MgSO_4_ and concentrated under reduced pressure. The crude product was purified by flash chromatography (EtOAc : hexane) to give the desired 1-substituted-1*H*-1,2,3,4-tetrazoles (ESI).†

## Results and discussion

### Catalyst characterization

The structure of 3 was characterized and confirmed by CHN, EDX, ^1^H-NMR, and ^13^C-NMR analyses (Experimental section). The average molecular weight for 4 was determined by GPC analysis, and was found to be ∼6000 g mol^−1^ with a satisfactory polydispersity index of 1.07 (dimensionless). Considering the mass of 408 g mol^−1^ for each monomeric unit (as shown in Scheme 3), therefore, the degree of polymerization of lattice polymer 3 is equal to 13 (considering the two end groups).

**Fig. 1:**
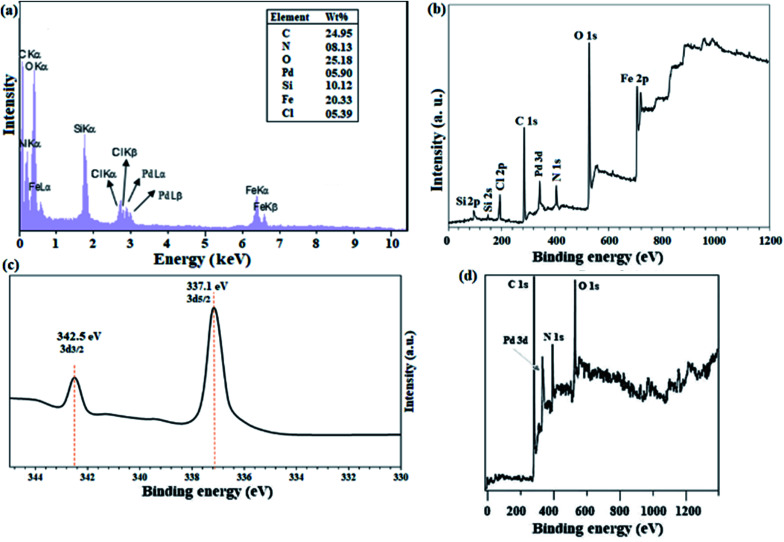
(a) EDX analysis, (b) overall survey and (c) Pd-3d high-resolution (normalized-energy corrected) XPS spectra of catalyst 9. (d) Overall survey XPS analysis of Pd(ii)-polysalophen 5.

ICP analysis from the catalyst revealed that 6 %wt Pd is present in the polymeric framework. Since 1.0 mmol of reactant was used in all reactions, the reactions were performed in the presence of 0.3 mol% Pd. Fig. 1a and b shows the results of EDX analysis and the XPS overall survey scan, in which the presence of all expected elements confirms the structure of the catalyst. XPS analysis of Pd(ii)-polysalophen also shows the complex formation well with the presence of a peak at 320 eV related to Pd binding energy (Fig. 1b). The presence of chlorine element in the EDX and XPS analyses well indicates the successful immobilization of Pd(ii)-polysalophen on the magnetic nanoparticles in full accordance with expectations and the proposed structure in Scheme 4. The presence of peaks at binding energies of 342 and 337 eV in the high resolution XPS analysis clearly shows that the Pd centers have a capacity of +2 (ref. 35) and no impurities were found arising from the presence of other species in the catalyst (Fig. 1c). Fig. 1d also shows the overall survey XPS analysis of polysalophen (5), confirming its structure with detection of the expected elements of Pd, C, N, O with high purity.

**Fig. 2:**
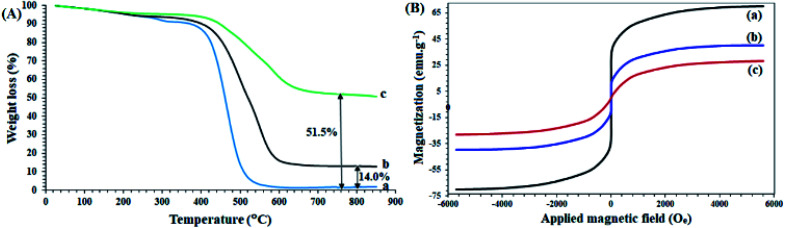
(A) TGA curves of (a) polysalophen, (b) Pd(ii)-polysalophen, and (c) Fe_3_O_4_@SiO_2_@Pd(ii)-polysalophen 9. (B) VSM curves of (a) Fe_3_O_4_, (b) Fe_3_O_4_@SiO_2_–Cl, and (c) Fe_3_O_4_@SiO_2_@Pd(ii)-polysalophen 9.

A study of the thermal behavior of the samples by TGA analysis provided useful information about the thermal resistance as well as the structure of the compounds, in agreement with other analyses. As shown in Fig. 2A-a, the thermal decomposition of the polysalophen ligand begins at a temperatures of about 220 °C, and continues with a gentle slope of up to 500 °C (Fig. 2A-a) and only 2% of the remaining weight is obtained. Coordination of Pd to polysalophen increases the thermal stability of the compound as expected (Fig. 2A-b). As shown in the figure, 14% of the remaining weight is generated up to 850 °C. This value can be attributed to the formation of PdO nanoparticles at the end of the thermal process, which confirms the formation of a stable Pd(ii)-polysalophen complex. Immobilization of complex 5 on Fe_3_O_4_@SiO_2_ nanoparticles caused greater thermal stability of the catalyst. According to the results, the thermal decomposition of the immobilized groups on the magnetic nanoparticles starts from a temperature of around 480 °C and continues with a much gentler slope up to 650 °C (Fig. 2A-c). Due to the absence of any degradable groups in the structure of Fe_3_O_4_@SiO_2_ (according to literature reports),^33,34^ the residual final weight (51.5%) can be directly attributed to the polysalophen complex immobilized on the magnetic nanoparticles. The reduction of the initial weight up to 120 °C is also related to the loss of water in the network structure of the compounds (for all three curves).^34,36^

Fe_3_O_4_ and Fe_3_O_4_@SiO_2_–Cl and catalyst 9 magnetic nanoparticles have superparamagnetic properties with coercivity equal to zero. As shown in Fig. 2B, Fe_3_O_4_ and Fe_3_O_4_@SiO_2_–Cl nanoparticles and catalyst 9 have saturation magnetizations of 70, 40 and 28 emu g^−1^, respectively. A significant drop in magnetization occurred after immobilization of Pd(ii)-polysalophen on Fe_3_O_4_ nanoparticles (Fig. 2B-c). Compared to the rate of magnetization loss due to silica coating (30 emu g^−1^), this 12 unit reduction can be directly attributed to the successful immobilization of Pd(ii)-polysalophen on magnetic nanoparticles, which by its diamagnetic effect dilutes the applied magnetic field and consequently reduces the magnetization of the Fe_3_O_4_ core.^37^

**Fig. 3:**
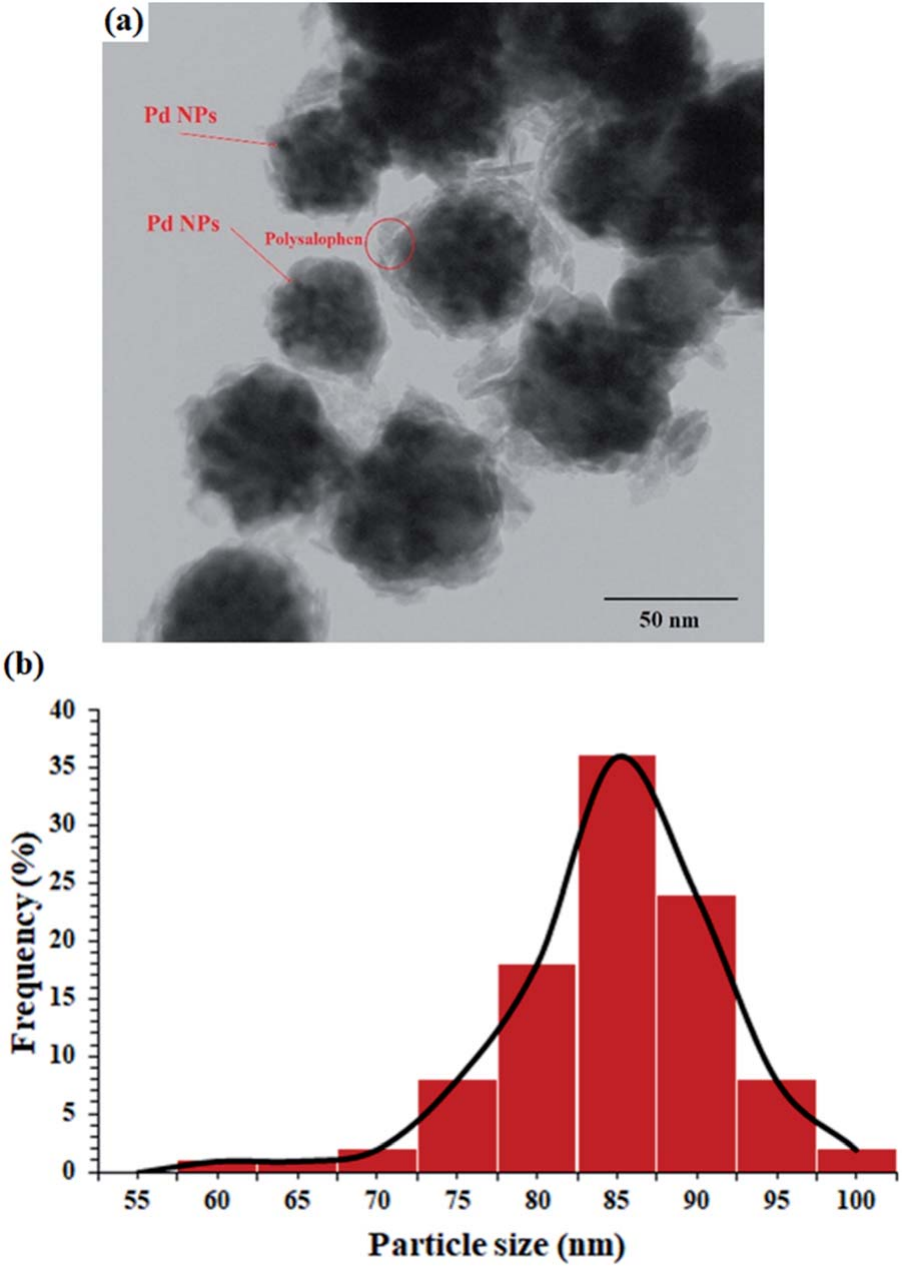
(a) TEM image and (b) DLS analysis of Fe_3_O_4_@SiO_2_@polysalophen-Pd(ii) 9.

The TEM image of the catalyst shows an average size of 75 nm for the particles (Fig. 3a). As well illustrated in the figure, the polymer chains appear as lighter regions as the phase is lighter than the core. In addition, very dark spots also represent the coordinated Pd particles in the structure of polysalophen.

**Fig. 4:**
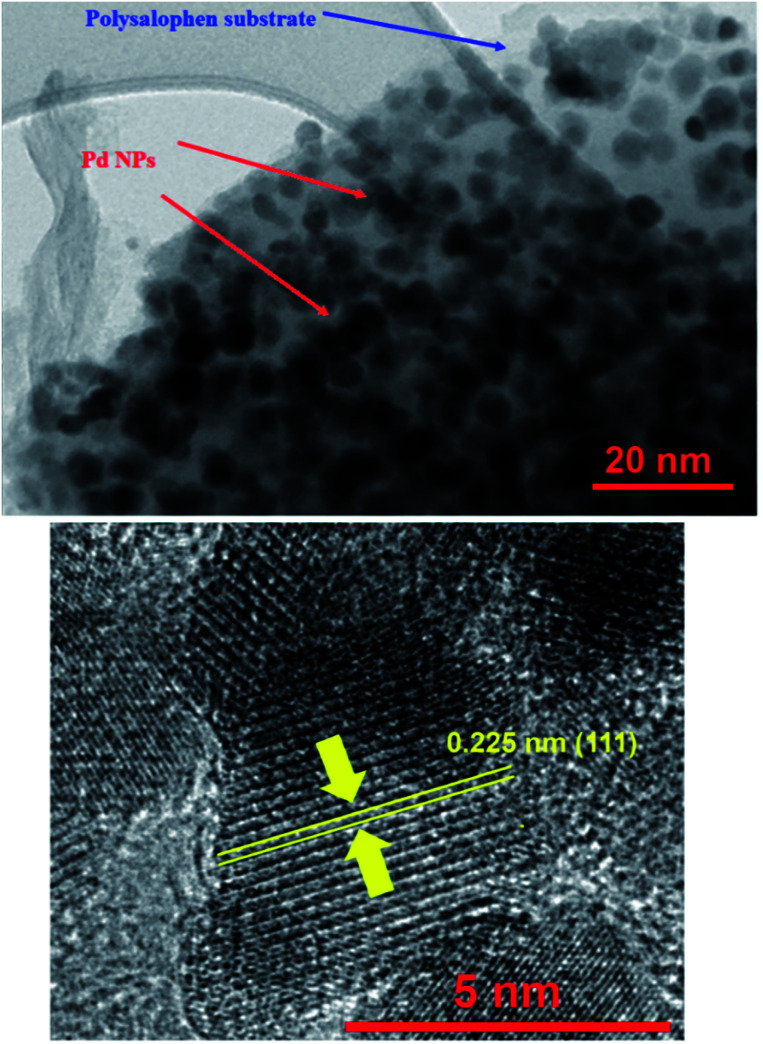
HRTEM images of Fe_3_O_4_@SiO_2_@polysalophen-Pd(ii) 9.

HRTEM analysis was also undertaken on the catalyst. As shown in the HRTEM image in Fig. 4, Pd NPs have been successfully loaded onto the polysalophen ligand with an average diameter size of 5 nm and an interplanar spacing of 0.223 nm related to the (111) planes of Pd, in agreement with the literature reports.^37–40^

DLS analysis also showed that most of the nanoparticles have an average size of 85 nm (Fig. 3b). This 10 nm difference in the size of TEM and DLS can be attributed to two factors:

(1) The difference in the nature of the two analyses is that in DLS, the laser light is deflected from the edges of the particles, somewhere farther from the actual particle size, according to published reports and (2) in accordance with the structure designed and proposed for the catalyst, *in situ* polymerization of salophen groups creates a phase with a lighter molecular weight around the magnetite nanoparticle and subsequently causes premature diversion of laser radiation in the DLS. Therefore, this difference of about 10 nm can be related to the dense chains around the nanoparticles, whereas in the TEM image, the brighter phases are quite clear.

**Fig. 5:**
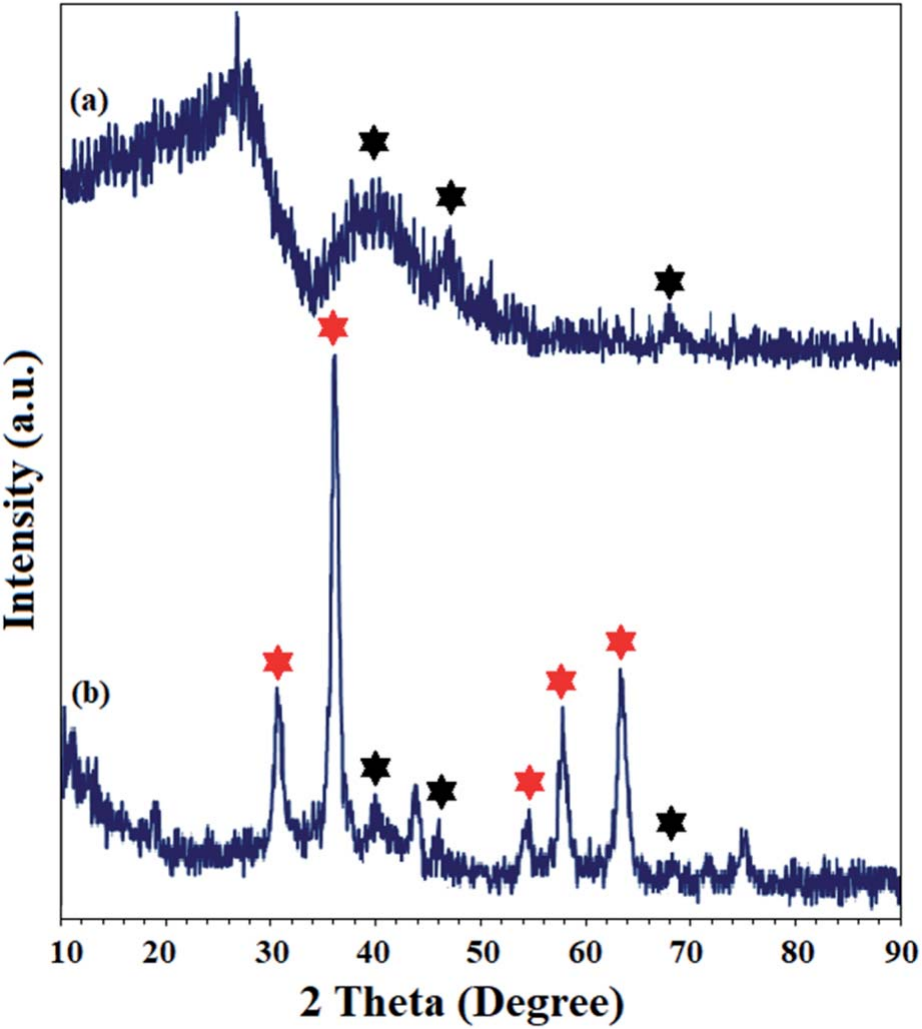
XRD patterns of (a) Pd(ii)-polysalophen and (b) Fe_3_O_4_@SiO_2_@Pd(ii)-polysalophen 9. Black and red stars represent the planes related to Pd (in Pd(ii)-polysalophen 5) and Fe_3_O_4_ crystal structures, respectively.

The crystal structure of Pd(ii)-polysalophen and the catalyst (9) were studied by X-ray diffraction analysis (Fig. 5). The three peaks appearing at angles 2*θ* = 40.2°, 46.0° and 68.1° correspond to planes (111), (200) and (220) in the Pd crystal structure, respectively, in full accordance with the reported diffraction patterns (Fig. 5a).^27^ The presence of peaks related to the crystal structure of Pd(ii)-polysalophen in the X-ray diffraction pattern of the catalyst at 2*θ* = 40°, 46° and 68° is strong evidence for its successful immobilization on the magnetic nanoparticles, so that no phase change or shift in the peak related to the crystal structure of magnetic nanoparticles occurred. These results reflect the non-alteration of the crystal structure of both phases of the magnetic nanoparticles as well as Pd(ii)-polysalophen after immobilization (Fig. 4b).

Surface characteristics of Fe_3_O_4_, Fe_3_O_4_@SiO_2_, Fe_3_O_4_@SiO_2_Cl, and catalyst 9 were evaluated by N_2_ adsorption–desorption isotherm analysis. According to the results, the specific surface areas for Fe_3_O_4_, Fe_3_O_4_@SiO_2_, Fe_3_O_4_@SiO_2_Cl, and catalyst 9 nanoparticles are equal to 478, 450, 398, and 352 m^2^ g^−1^ (Table 1). At each stage of functionalization, the specific surface area of the magnetite nanoparticles is reduced, which reflects the immobilization of different groups on the surface of the nanoparticles. In addition, the porosity volume also decreased with the reduction of the surface area, at each stage of immobilization of various groups. Significantly, there is a significant reduction in the specific surface area and porosity after immobilization of Pd(ii)-polysalophen on Fe_3_O_4_@SiO_2_–Cl nanoparticles, which is a good indication of its successful immobilization, which is quite comparable to the results obtained from the silica coating on the nanoparticles (Table 1, entry 4). Subsequently, the porosity increased at each stage of functionalization, and after immobilization of the Pd(ii)-polysalophen, a significant decrease in agreement with its immobilization was observed (Table 1, entry 4).

**Table 1:** Surface characteristics of Fe_3_O_4_, Fe_3_O_4_@SiO_2_ Fe_3_O_4_@SiO_2_–Cl, and Fe_3_O_4_@SiO_2_@polysalophen-Pd(ii) (9)

Entry	Sample	Specific surface area (m^2^ g^−1^)	Pore volume (cm^3^ g^−1^)	Average pore radius (nm)
1	Fe_3_O_4_	478	0.805	1.255
2	Fe_3_O_4_@SiO_2_	450	0.782	1.788
3	Fe_3_O_4_@SiO_2_–Cl	398	0.750	1.800
4	Catalyst 9	352	0.700	1.865

### Optimization of reaction parameters

**Fig. 6:**
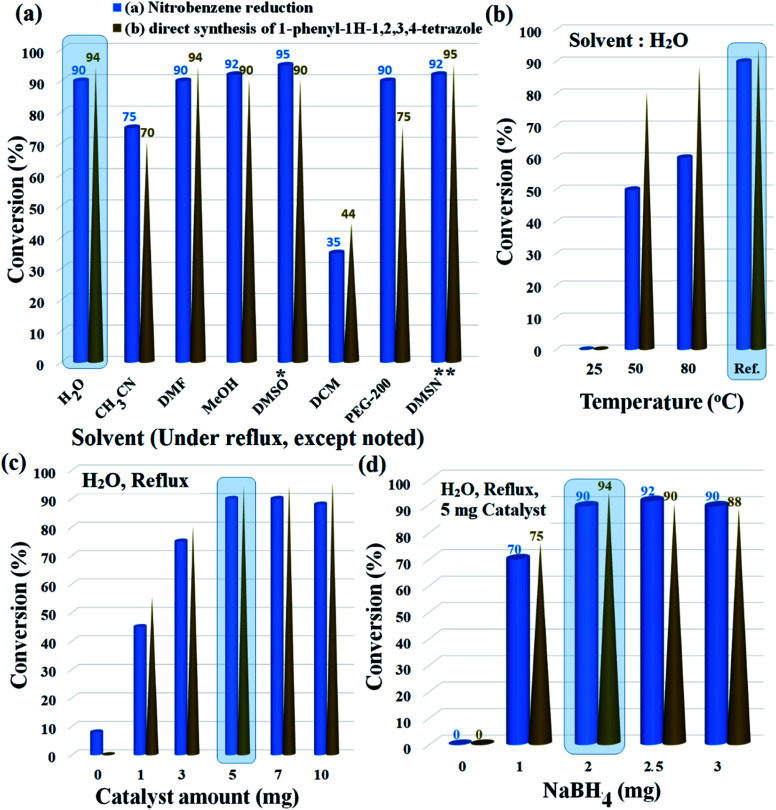
Influence of (a) solvent, (b) temperature, (c) catalyst amount, and (d) NaBH_4_ amount on the reduction of nitrobenzene and direct transformation of nitrobenzene to the corresponding 1-phenyl-1*H*-1,2,3,4-tetrazole. *General reaction conditions (except as noted in each figure):*
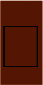
*Nitrobenzene (1.0 mmol), NaBH*_*4*_*(2.0 mmol), H*_*2*_*O (2.0 mL), catalyst*9*(5 mg, 0.3 mol% Pd), reflux, 60 min.*
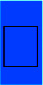
*Nitrobenzene (1.0 mmol), triethyl orthoformate (1.2 mmol), NaN*_*3*_*(1.5 mmol), NaBH*_*4*_*(2.0 mmol), H*_*2*_*O (2.0 mL), catalyst*9*(5 mg, 0.3 mol% Pd), reflux, 90 min.* * 120 °C. ** Dimethyl sulfone, 120 °C. *In each experiment, the premium conditions are shown shaded in blue, that was used for the next run*.

The reduction of nitrobenzene and its following transformation to the corresponding 1-phenyl-1*H*-1,2,3,4-tetrazole were chosen as two separate model reactions to optimize temperature, catalyst amount, reducing agent, and solvent parameters. The best choice was distilled water with 90% and 94% conversion for the preparation of aniline and 1-phenyl-1*H*-1,2,3,4-tetrazole, respectively. Although solvents such as DMSO, DMSN, and MeOH produced higher efficiencies for aniline, the efficiencies for tetrazole were lower than for water. In addition, parameters such as environmental friendliness, susceptibility, and availability also led to the selection of water as the premium solvent for the reactions. Another noteworthy point was the selectivity above 98% for the reduction of nitrobenzene in water; DMSO, DMSN and DMF solvents also produced similar selectivity (above 98%). Due to the high efficiency created by polar solvents with high dielectric constant, the results suggest a mechanism involving polar intermediates. As shown in Fig. 6a and b, water solvent under reflux conditions created the highest possible efficiency with 90% and 94% efficiencies for aniline and 1-phenyl-1*H*-1,2,3,4 tetrazole, respectively.

Lowering the temperature to 50 °C reduced the efficiencies by up to 50% and 80% for aniline and 1-phenyl-1*H*-1,2,3,4-tetrazole, respectively. No efficiency at room temperature was observed for any of the products. Another effective parameter was the amount of catalyst, which in the optimal amount of 5 mg, resulted in the highest efficiency for both reactions. Higher (up to 10 mg) and lower values were unfavorable for both reactions. In the absence of the catalyst, only 8% efficiency was observed for aniline (0% efficiency for tetrazole), which reflects the catalytic activity of Fe_3_O_4_@SiO_2_@polysalophen-Pd^(II)^ (9) to reduce nitrobenzene and prepare tetrazole (Fig. 6c).

In addition, since the loading amount of metal nanoparticles on the composite support has an influence on the particle size as well as their catalytic activity, the effect of the Pd loading value on the complex was also studied. In order to investigate the effect of different amounts of nanoparticle loading in the catalyst, by changing the amount of Pd salt used in the preparation of the polysalophen complex (Scheme 3), different percentages of loaded Pd were obtained. Pd complexes of polysalophen with different amounts of Pd were immobilized on Fe_3_O_4_@SiO_2_ nanoparticles. The loading of Pd nanoparticles in the catalyst was measured by EDX analysis (5-point mean) as well as ICP analyses. DLS analysis was also performed to determine the size of the catalyst (ESI, Tables S1 and S2†).

Table S1† shows the results of ICP, EDX and DLS analyses. For a better comparison, the synthesized catalyst in this work with 6 %wt Pd is also given in Table S1† (entry 2). Increasing the amount of Pd nanoparticles increased the average particle size (by DLS analysis). The average nanoparticle size increased from 82 nm with 2.22 %wt Pd to 94 nm with 18 %wt Pd. A noteworthy point is the high loading capacity of polysalophen complex compared to Pd, so that different loading percentages of 2.22, 6.06, 10.11, 14.16, and 17.19 wt% were prepared. The catalytic activity of the catalysts was studied in three reactions: reduction of nitrobenzene to aniline, direct conversion of nitrobenzene and conversion of aniline to 1-substituted-1*H*-1,2,3,4-tetrazoles. Based on the results of ICP and EDX, catalysts were defined as: Cat. 9/0.12 mol% Pd; Cat. 9/0.3 mol% Pd; Cat. 9/0.6 mol% Pd; Cat. 9/0.8 mol% Pd; Cat. 9/1.0 mol% Pd.

Increasing the amount of Pd nanoparticles to 0.8 mol% had no significant effect on the reaction efficiency, and only the reduction efficiency of nitrobenzene to aniline showed a slight increase (Table S2,† row 4). The yield decreased by decreasing the Pd Ps at 5 mg of catalyst, in accordance with the results of optimizing the amount of catalyst (Fig. 5c), where by reducing the weight of the catalyst (corresponding to the reduction in mol% Pd), the reaction efficiency decreased significantly. It seems that increasing the amount of Pd nanoparticles blocks some active sites (due to agglomeration of Pd nanoparticles in the catalyst framework due to increasing their number) and consequently reduces the catalytic activity. Although due to the increase in the amount of nanoparticles in the catalyst, its surface charge increases and repulsion is created, causing better dispersion of nanoparticles, but increasing the number of Pd nanoparticles in the structure of polysalophen blocks active sites and acts in the opposite direction. Therefore, increasing the amount of Pd nanoparticles loaded on the magnetic substrate had a similar result to the effect of the weight of the catalyst in the reactions. On the other hand, reducing the amount of catalyst also caused a decrease in efficiency in all 3 reactions with a process similar to the results of reducing the weight of the catalyst in the optimized experiments.

Finally, the stoichiometric amount of NaBH_4_ as a reducing agent was studied in the presence of 5 mg of catalyst and water solvent under reflux conditions. The efficiency reaches a maximum in a stoichiometric amount of twice that of nitrobenzene. As shown in Fig. 6d, stoichiometric values higher and lower for NaBH_4_ were consistent with decreased aniline efficiency and the subsequent preparation of tetrazole compounds. Considering the correlation between nitrobenzene reduction efficiency and also the direct conversion of nitrobenzene to tetrazole, therefore, the results show good preparation of tetrazoles through amino intermediates and subsequent domino reaction. Another proof of this claim is the absence of tetrazole product in the absence of NaBH_4_ in the direct conversion of nitrobenzene to tetrazole (Fig. 6d).

For a better understanding, the results of optimization experiments only in water solvent (as the optimal solvent) are summarized in Table 2.

**Table 2:** Summary of the results of optimizing the parameters of the reduction of nitrobenzene^a^ and direct transformation of nitrobenzene to the corresponding 1-phenyl-1*H*-1,2,3,4-tetrazole in water solvent^b^

Entry	Solvent	*T*. (°C)	Cat. amount (mg)	NaBH_4_	Product			
					Aniline		1-Phenyl-1*H*-1,2,3,4-tetrazole	
					Time (min)	Yield (%)	Time (min)	Yield (%)
1	H_2_O	25^c^	5	2	60	0	90	0
2	H_2_O	50	5	2	60	50	90	80
3	H_2_O	80	5	2	60	60	90	88
4	H_2_O	Reflux	5	2	60	90	90	94
5	H_2_O	Reflux	0	2	60	8	90	0
6	H_2_O	Reflux	1	2	60	45	90	55
7	H_2_O	Reflux	3	2	60	75	90	80
8	H_2_O	Reflux	7	2	60	90	90	94
9	H_2_O	Reflux	10	2	60	88	90	95
10	H_2_O	Reflux	5	0	60	0	90	0
11	H_2_O	Reflux	5	1	60	70	90	75
12	H_2_O	Reflux	5	2.5	60	92	90	90
13	H_2_O	Reflux	5	3	60	90	90	88

a Reaction conditions: nitrobenzene (1.0 mmol), NaBH_4_ (2.0 mmol), H_2_O (2.0 mL), catalyst 9 (5 mg, 0.3 mol% Pd), reflux, 60 min.

b Nitrobenzene (1.0 mmol), triethyl orthoformate (1.2 mmol), NaN_3_ (1.5 mmol), NaBH_4_ (2.0 mmol), H_2_O (2.0 mL), catalyst 9 (5 mg, 0.3 mol% Pd), reflux, 90 min.

c Room temperature.

### Catalytic activity

**Scheme 5:**
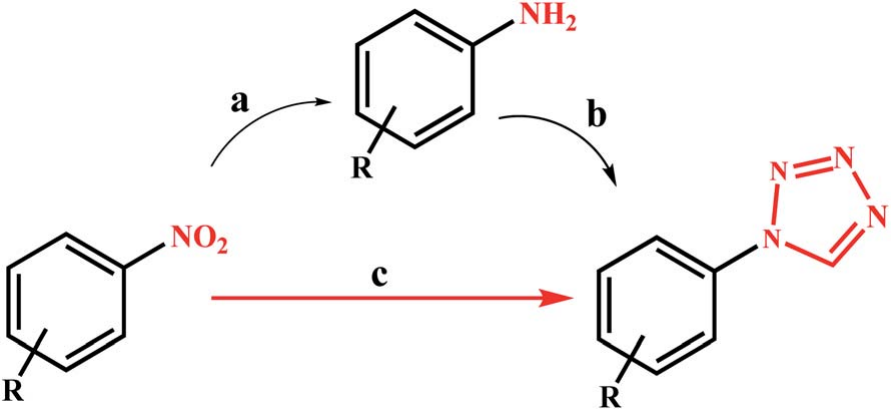
Reduction of nitroarenes and domino preparation of 1-substituted-1*H*-1,2,3,4-tetrazoles catalyzed by Fe_3_O_4_@SiO_2_@polysalophen-Pd(ii) 9. (a) Nitroarene (1.0 mmol), NaBH_4_ (2.0 mmol), H_2_O (2.0 mL), catalyst 9 (5 mg, 0.3 mol% Pd), reflux. (b) amine (1.0 mmol), triethyl orthoformate (1.2 mmol), catalyst 9 (5 mg, 0.3 mol% Pd), NaN_3_ (1.5 mmol), H_2_O (2.0 mL), reflux. (c) Nitroarene (1.0 mmol), triethyl orthoformate (1.2 mmol), NaN_3_ (1.5 mmol), NaBH_4_ (2.0 mmol), H_2_O (2.0 mL), catalyst 9 (5 mg, 0.3 mol% Pd), reflux.

Under optimal conditions, various derivatives of nitro compounds were reduced to the corresponding amines. Also, the direct conversion of nitroarenes and also the conversion of amines to the corresponding tetrazole products were studied with Fe_3_O_4_@SiO_2_@polysalophen-Pd^(II)^ (9). Scheme 5 shows the conditions used for each conversion.

Table 2 shows the reduction of a wide range of nitroarenes in the presence of Fe_3_O_4_@SiO_2_@polysalophen-Pd^(II)^ (9). As shown in the table, excellent efficiency is obtained for almost all nitro compounds.

Most importantly, the method has high selectivity, so that a selectivity of over 95% is achieved in a period of 45–90 minutes for the nitroarene reduction. This advantage is very important due to the formation of various products in the resultant reduction of a nitrobenzene (Scheme 2). The electronic effects were also not very significant and the efficiency for nitroarenes with electron-donor and electron-withdrawing groups was almost the same (Table 3). However, nitroarenes containing electron-withdrawing groups were reduced in a shorter time than those with electron-donor groups. Nevertheless, high to excellent selectivity was obtained for all derivatives (Table 3).

**Table 3:** Reduction of nitroarenes over Fe_3_O_4_@SiO_2_@polysalophen-Pd(ii) 9 in the presence of NaBH_4_^a^^,^^b^

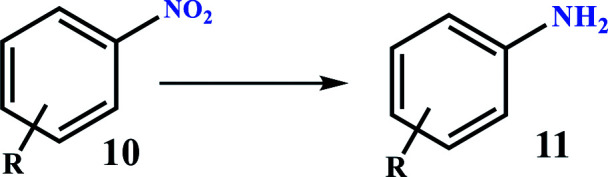						
Entry	R	P.	*t* (min)	*Y*.^c^ (%)	*S*. (%)	MP (°C)
1	H	11a	60	96	99	Oil
2	4-MeO	11b	80	94	98	59–62
3	4-OH	11c	90	76	96	184
4	4-Me	11d	85	90	99	38–40
5	4-Cl	11e	80	92	97	70–72
6^d^	4-CN	11f	65	92	95	88
7	2-Pyridyl	11g	65	90	99	155
8^c^^,^^e^	3-NO_2_	11h	50	98	97	61–63
9	4-COMe	11i	50	95	96	104–106
10	4-CO_2_Me	11j	45	97	96	185
11	3-NH_2_-4-Cl	11k	70	95	98	63–65

a Reaction conditions: see Scheme 5. Nitroarene (1.0 mmol).

b Definition of the variables: P. = product; *t* = time; *Y*. = isolated yield; S. = selectivity.

c Isolated yield.

d No reduction for cyano group (preparation of *p*-phenylenediamine) was observed.

e Catalyst: 10 mg.

Table 4 shows the results of the direct conversion of nitroarenes to 1-substituted-1*H*-1,2,3,4-tetrazoles. Also, in parallel reactions to compare the catalytic process, this conversion was performed from the corresponding amines. As shown in Table 3, there is no significant difference in conversion reaction times for amines in comparison with nitroarenes. These results indicate that the nitro groups participate in the tetrazole ring formation reaction immediately after reduction on the catalyst surface. This also contributes to the further development of the reduction of nitro compounds and the subsequent preparation of tetrazole compounds. As with the reduction of nitro compounds, good to excellent efficiency was achieved for the direct conversion of nitro compounds as well as the aromatic conversion of amines to the corresponding tetrazoles.

**Table 4:** Domino and traditional preparation of 1-substituted-1*H*-1,2,3,4-tetrazoles from nitro and amine, respectively, catalyzed by Fe_3_O_4_@SiO_2_@polysalophen-Pd(ii)^a^^,^^b^

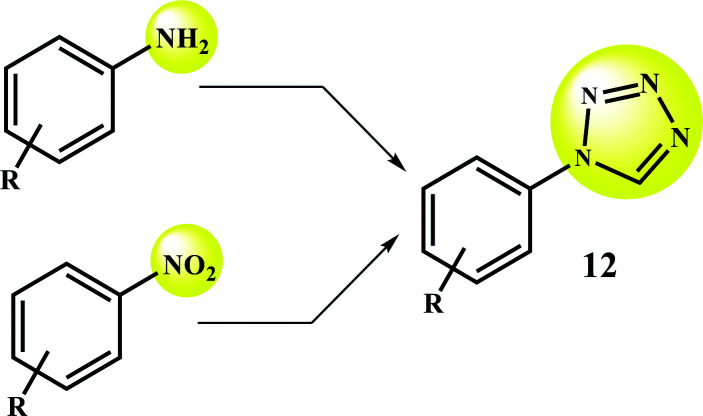							
Entry	R	P.	From amine		From nitro		MP (°C)^[Lit.]^
			*t* (min)	*Y*.^c^ (%)	*t* (min)	*Y*.^c^ (%)	
1	H	12a	75	97	90	98	64–65 (ref. 42)
2	4-MeO	12b	60	96	80	96	114–115 (ref. 42^)^
3	4-OH	12c	90	96	130	98	185–187 (ref. 13)
4	4-Me	12d	90	95	120	96	94–96 (ref. 13)
5	4-Cl	12e	110	95	150	98	157–158 (ref. 42)
6	4-CN	12f	120	94	150	90	177–179 (ref. 13)
7	2-Pyridyl	12g	80	90	120	92	80–82 (ref. 13)
8	3-NO_2_	12h	140	95	160	95	106–108 (ref. 13)

a For reaction conditions see Scheme 5. Amine (1.0 mmol), nitrobenzene (1.0 mmol).

b Definition of the variables: P. = product; *t* = time; *Y*. = isolated yield.

c Isolated yield.

### Control experiments

In order to evaluate and confirm the uniqueness of the catalyst activity in nitrobenzene reduction as well as the direct preparation of 1-phenyl-1*H*-1,2,3,4-tetrazole from nitrobenzene, different control reactions in the presence of different catalyst components were studied. According to the results summarized in Table 4, the catalyst activity for both products is well demonstrated. The compounds including Fe_3_O_4_, Fe_3_O_4_@SiO_2_ and polysalophen 3 did not produce any efficiency for tetrazole (Table 5, entries 1–3). Only very small efficiencies of 8% and 16% were observed for the reduction of nitrobenzene to aniline in the presence of Fe_3_O_4_ and Fe_3_O_4_@SiO_2_, respectively. The absence of catalytic activity of these compounds for the preparation of tetrazole can be directly attributed to the absence of any coordinated transition metal; because Pd(ii)-polysalophen produced 80 and 65% efficiency for aniline and 1-phenyl-1*H*-1,2,3,4-tetrazole, respectively (Table 5, entries 4 and 5). However, the efficiency was lower than that of the catalyst (9), which could be attributed to the immobilization of Pd(ii)-polysalophen on the magnetite nanoparticles. Immobilization of Pd(ii)-polysalophen on the magnetite provides a cohesive lattice structure with a high surface-to-volume ratio (according to the proposed structure for the catalyst in Scheme 4) that causes the compounds to penetrate into the catalyst structure in an aqueous medium, and after the reaction, they are separated from the surface of the catalyst, in full agreement with the suggested mechanisms that water has been used as a solvent in the organic reactions.^41–44^

**Table 5:** Screening of catalytic activity of the catalyst component toward nitrobenzene reduction^a^ and the preparation of 1-phenyl-1*H*-1,2,3,4-tetrazole^b^ Fe_3_O_4_@SiO_2_@polysalophen-Pd(ii) as a control experiment

Entry	Catalyst	Product			
		Aniline		1-Phenyl-1*H*-1,2,3,4-tetrazole	
		Time (min)	Conversion (%)^c^	Time (min)	Conversion (%)^c^
1	Fe_3_O_4_	60	8	90	N.R.
2	Fe_3_O_4_@SiO_2_	60	16	90	N.R.
3	Polysalophen 3	60	N.R.	90	N.R.
4	Pd(OAc)_2_	60	5	90	10
5	Pd(ii)-polysalophen complex	60	80	90	65
6^d^	Pd(ii)-polysalophen complex + Fe_3_O_4_@SiO_2_	60	80	90	65

a Nitrobenzene (1.0 mmol), NaBH_4_ (2.0 mmol), H_2_O (2.0 mL), catalyst (5 mg), reflux.

b Nitrobenzene (1.0 mmol), triethyl orthoformate (1.2 mmol), NaN_3_ (1.5 mmol), NaBH_4_ (2.0 mmol), H_2_O (2.0 mL), catalyst (5 mg), reflux.

c GC.

d The reaction was performed in the presence of a physical mixture of Pd(ii)-polysalophen complex (0.5 g) and Fe_3_O_4_@SiO_2_ (0.5 g).

In order to investigate this phenomenon, in another control test, the catalytic activity of a physical mixture of Pd(ii)-polysalophen and Fe_3_O_4_@SiO_2_ was studied (Table 5, entry 6).

The results clearly provided similar efficiency to Pd(ii)-polysalophen. The results not only show the successful and effective immobilization of Pd(ii)-polysalophen on magnetic nanoparticles, but also show that the structure of the catalyst provides a suitable environment for reduction and tetrazole conversions in an aqueous medium.

### Reaction mechanism

**Scheme 6:**
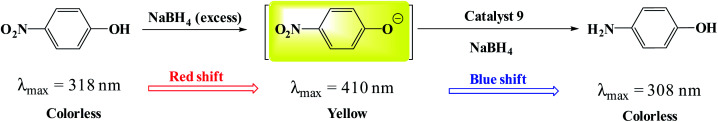
A schematic view of the reduction of 4-nitrophenol catalyzed by Fe_3_O_4_@SiO_2_@polysalophen-Pd(ii) (9).^47^

**Fig. 7:**
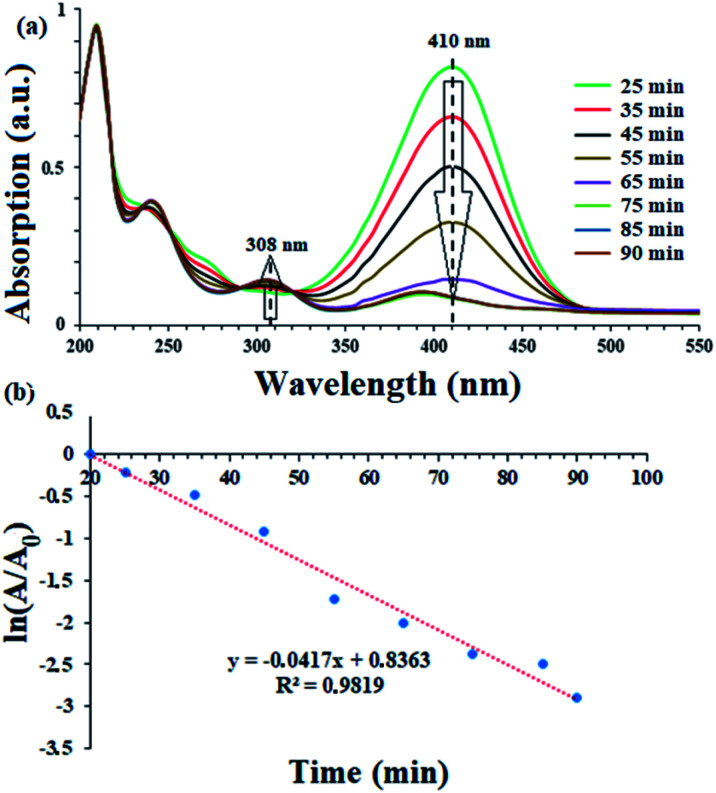
(a) Consecutive UV-Vis spectra for 4-nitrophenol reduction to the corresponding 4-aminophenol acquired after *t*_0_ = 25 min of addition of NaBH_4_. The spectra were recorded every 10 minute until full reduction. For greater clarity, the recorded spectra before *t*_0_ have been removed. *Reaction conditions: NaBH*_*4*_*(2.0 mmol), H*_*2*_*O (2.0 mL), catalyst*9*(5 mg, 0.3 mol% Pd), reflux*. (b) Plot of ln(*A*/*A*_0_) *versus* time for the reduction of 4-nitrophenol for a period of 25–90 minutes (after induction time).

To evaluate the activity of the catalyst in the reduction of nitro compounds, the reduction of 4-nitrophenol was selected as a model reaction. As shown in Scheme 6, the presence of the OH group increases the length of the conjugate system during the reduction process and the subsequent red shift to 410 nm. 4-Aminophenol also has an absorption band at 308 nm.^45,46^ Therefore, by continuously studying the electronic spectra of the 4-nitrophenol reduction reaction mixture, the reduction process can be studied. Fig. 7a shows the results of continuous electronic spectra from 4-nitrophenol reduction in the presence of catalyst (9). At the beginning of the reaction, 4-nitrophenol species are converted to phenolate anions in the presence of NaBH_4_, which has an absorption band at 410 nm. After reduction of the nitro group, the absorption decreases and a new absorption appears at 308 nm related to 4-aminophenol. Fig. 7a shows the presence of both absorption bands simultaneously in the last 35 minutes of the reaction. This time is related to the induction time in the presence of NaBH_4_ and represents the diffusion time required to adsorb 4-nitrophenol on the surface of the catalyst (as shown in the proposed mechanism), and then the reduction takes place.^46,47^ As the reaction progresses and the phenolate intermediate is consumed, its absorption intensity at 410 nm decreases, and the absorption intensity at 308 nm increases due to the formation of 4-aminophenol. To confirm this phenomenon, in a control reaction, 4-nitrophenol reduction was tested in the absence of the catalyst and in the presence of NaBH_4_. The results showed no efficiency for 4-aminophenol and only the absorption band at 318 nm for 90 min belonging to 4-nitrophenol was observed. The results well showed the reduction process of 4-nitrophenol over time. In addition, with these results, the reaction kinetics can be studied spectrophotometrically. According to the results, the induction time is equal to 25 minutes and therefore the reduction of the nitro group takes place after this time (*t*_0_ = 25 min).

The results show that the reduction of nitro compounds follows first-order kinetics.^48,49^ In order to investigate the kinetics of the 4-nitrophenol reduction, ln(*A*/*A*_0_) was plotted in Fig. 7b in terms of time according to the electronic spectra (UV-Vis) obtained.

Accordingly, the recorded absorption at 408 nm was used over a period of 25 to 90 minutes. With constant consideration of NaBH_4_, the rate constant for the reaction for 4-nitrophenol reduction can be calculated with the following eqn (2):^46^2
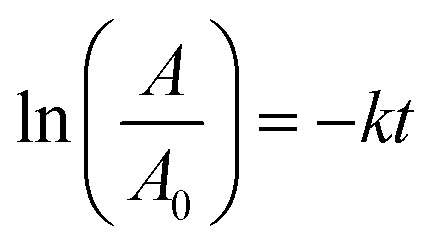
where *A* is the absorption of 4-nitrophenol at time *t* (sec.), *A*_0_ is the initial absorbance of 4-nitrophenol. According to the equation, the rate constant for the reduction was calculated as 0.0007 s^−1^.

**Scheme 7:**
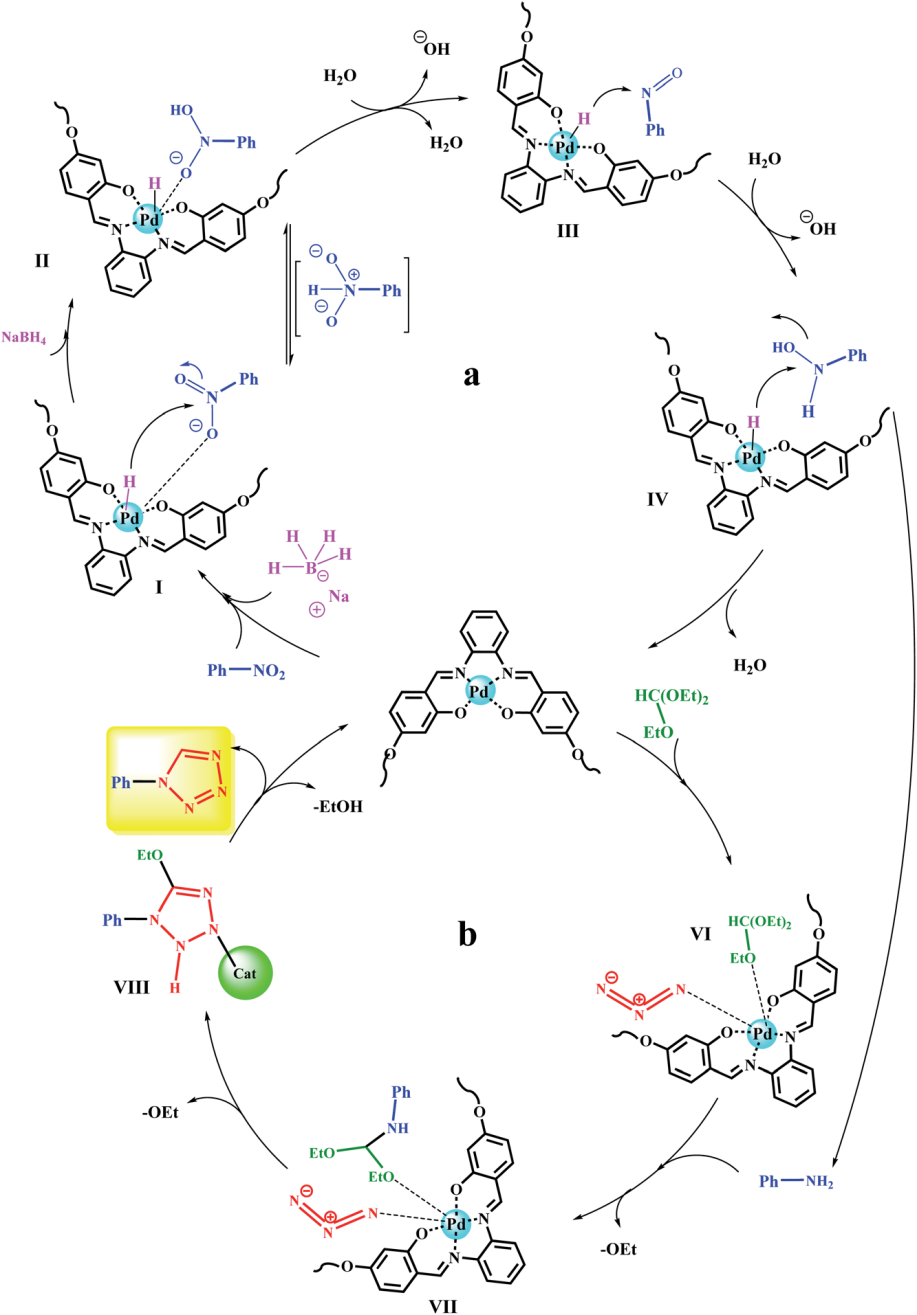
Plausible reaction mechanisms for (a) reduction of nitroarenes and (b) their subsequent transformation to 1-substituted-1*H*-1,2,3,4-tetrazoles catalyzed by Fe_3_O_4_@SiO_2_@polysalophen-Pd(ii).

Scheme 7 shows the reduction of nitrobenzene and its subsequent conversion to tetrazole in two proposed mechanisms. Initially, the nitro and reducing agent (NaBH_4_) can be adsorbed on the catalyst surface.^45,50^ The hydride is then transferred to the nitro group through four consecutive intermediates (I, II, III, IV). The presence of water and NaBH_4_ at each step causes the effective transfer of hydride to the nitro compound and the removal of the produced hydroxyl group as a water molecule.^51–53^

**Fig. 8:**
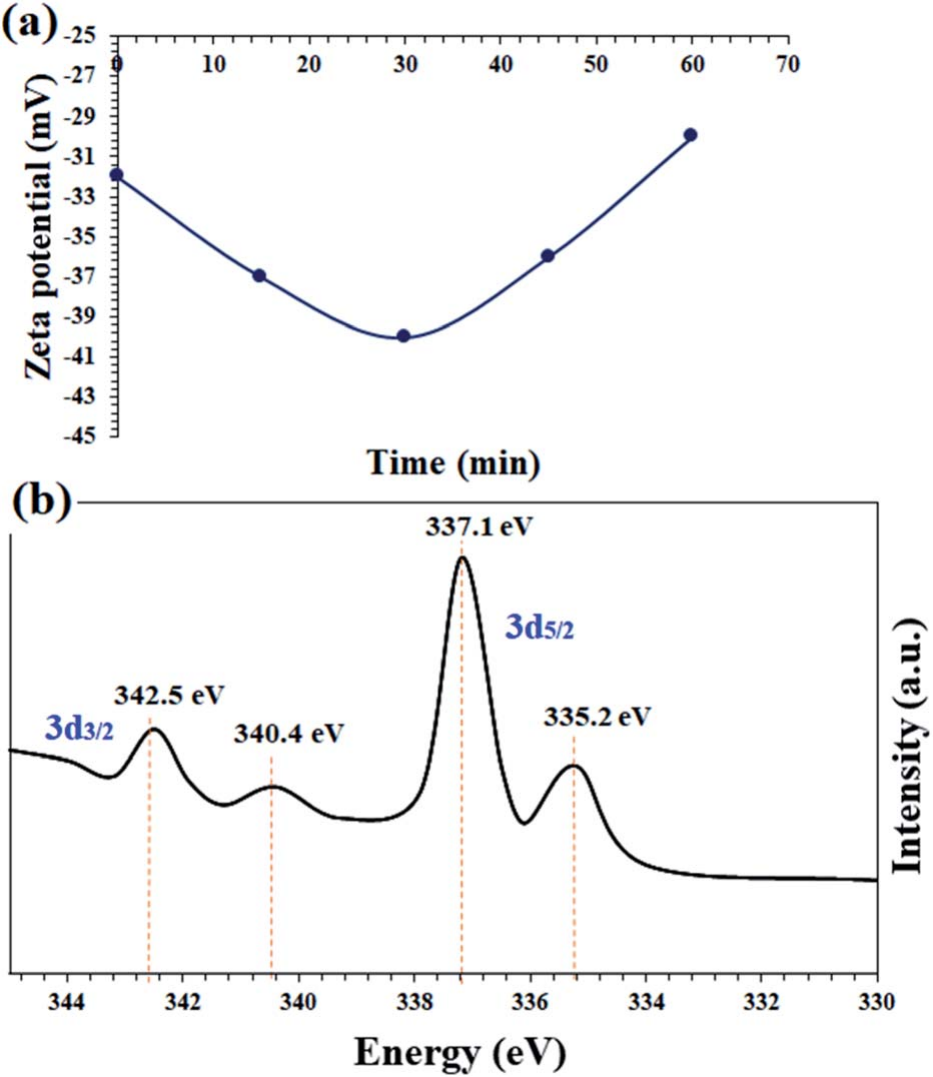
(a) Monitoring the zeta potential of the catalyst in terms of the reaction time in the reaction mixture of nitrobenzene reduction and (b) *in situ* high resolution Pd-3d (normalized, energy-corrected) XPS analysis of the recovered catalyst 9 after 30 min of the nitrobenzene reduction reaction.

In order to confirm the proposed mechanism, the catalyst surface charge was recorded by zeta potential analysis at 15, 30 and 45 minutes and also at the end of the nitrobenzene reduction reaction. Fig. 8a shows the results of this analysis. The freshly prepared catalyst has a zeta potential of −32, which is completely consistent with the prepared catalysts based on the immobilization of a transition metal complex on magnetic nanoparticles.^54,55^ After the reaction, the zeta potentials of the catalyst reach −37 and −40 for 15 and 35 minutes, respectively. This amount of negativity is due to the adsorption of BH_4_^−^ on the surface of the catalyst and the subsequent reduction of nitrobenzene. After the reaction and the consumption of NaBH_4_, the zeta potential goes to positive values and at the end of the reaction has almost the same initial potential equal to −30. These results well show the transfer of hydride ions from the catalyst to nitrobenzene.

*In situ* XPS analysis was also performed at 30 min of the nitrobenzene reduction reaction. The results well show the presence of Pd(0) species in the respective binding energies at 342.5 eV (Pd 3d_3/2_) and 335.2 eV (Pd 3d_5/2_),^35^ along with Pd^2+^ species (Fig. 8b). The results are in complete agreement with the proposed mechanism, in which Pd centers play the role of hydride transfer, thereby converting Pd^2+^ species to Pd(0).

Upon reducing the nitro to amine, in the next step, a domino reaction is performed to prepare tetrazoles in the presence of triethyl orthoformate. As shown in Scheme 7, triethyl orthoformate and azide ions (intermediate VI) can be adsorbed on the catalyst surface. Intermediate VII is formed by the nucleophilic attacks of the previously produced amine on triethyl orthoformate, and the subsequent removal of an ethanol molecule. By a 3 + 2 ring-formation reaction, a tetrazole ring is formed and by removing another ethanol molecule, the desired tetrazole product is obtained. The catalyst also returns to the cycle after this step.

### Reusability

**Fig. 9:**
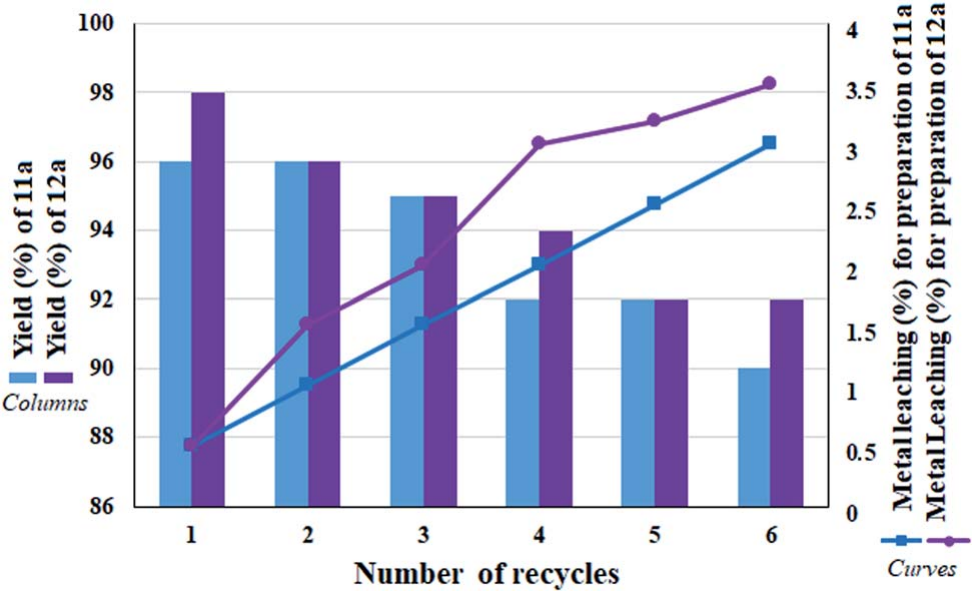
Recycling studies and measurement of leaching amount of heterogeneous catalyst 9 over the preparation of 11a and 12a under optimized conditions.

The ability to recover a heterogeneous catalyst is very important in that (1) some reagents in the reaction mixture have the ability to inactivate the catalyst (also in the presence of some functional groups) and (2) in catalytic systems containing a transition metal, there is a possibility of metal leaching and subsequent reaction progress in the presence of the lost metal.^31,33,34^ From this point of view, due to the presence of NaBH_4_ in the reaction, metal leaching is very undesirable for the catalyst. Hence, the recoverability of the catalyst was studied for 6 consecutive runs in the direct conversion reaction of nitrobenzene to tetrazole as well as the reduction of nitrobenzene to aniline. In addition, in each cycle, after recovery of the catalyst, the remaining solution was studied to determine the amount of leaching by ICP analysis. Fig. 9 shows the results of recovery experiments for both reactions. According to the results, in each cycle, the amount of efficiency drop for both reactions was very small, and after the sixth cycle, the efficiency dropped to 90% and 92% (equivalent to 6% efficiency drop after 6 consecutive cycles for both reactions) for 11a and 12a, respectively. This amount of efficiency drop was correlated with the amount of metal leaching, so that in each cycle, some metal leaching was detected in the remaining solution. The amount of Pd leaching after the sixth cycle for 11a and 12a was determined to be 3% and 3.5%, respectively. This amount of leaching is acceptable and reasonable for heterogeneous catalysts. Also it has not caused a sharp drop in yield of the products. Another noteworthy point is that despite the small amount of leaching observed in the first cycle, no change in the reaction efficiency was observed with respect to the freshly prepared catalyst, which reflects the stability and high activity of the catalyst.

**Fig. 10:**
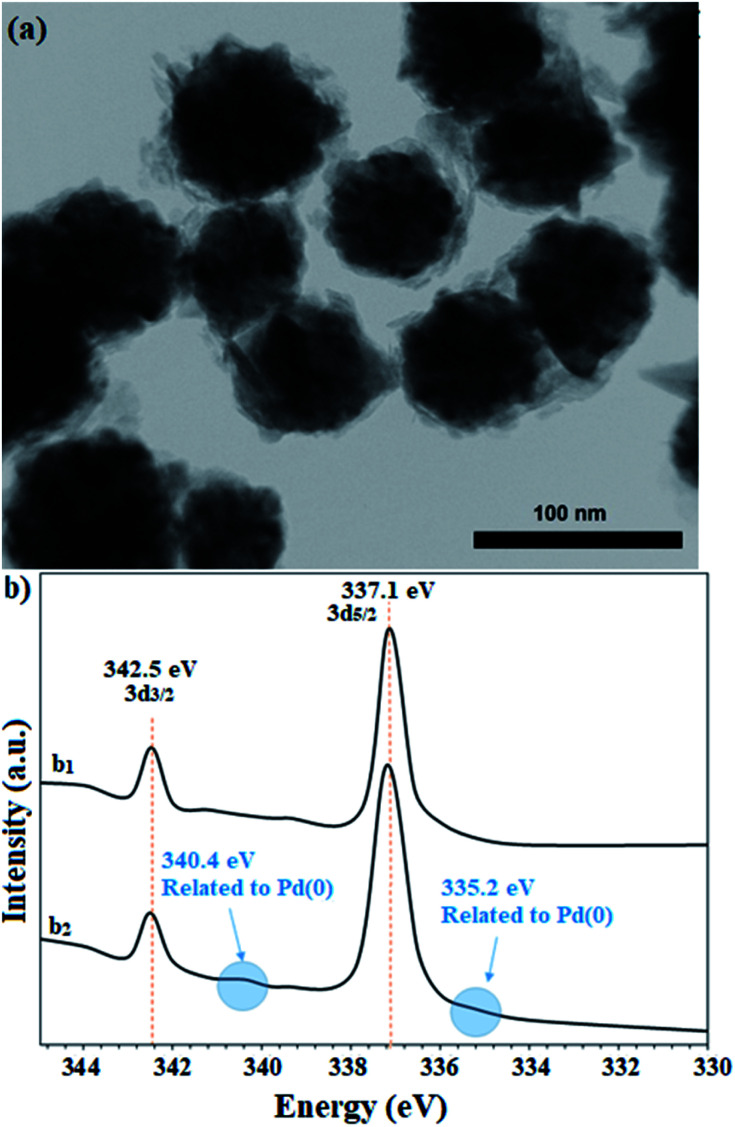
(a) TEM image and (b) high resolution XPS-Pd-3d (normalized, energy-corrected) analysis of (b_1_) the freshly prepared and (b_2_) the recovered catalyst 9 after the 6^th^ run.

The recovered catalyst after the sixth cycle was studied by TEM analysis to evaluate its structure and morphology. Fig. 10a shows the TEM image of the recovered catalyst after the sixth cycle, in which no trace of agglomeration or change in particle morphology can be observed. In addition, compared to the TEM image of the freshly prepared catalyst, the unchanged particles have an average diameter of 75 nm. Due to the nature of the reduction reaction and the presence of NaBH_4_ as the reduction agent in the reactions, XPS analysis was also conducted on the recovered catalyst after the sixth cycle. As shown in Fig. 10b, the Pd centers have maintained their oxidation state capacity as +2 as in the freshly prepared catalyst. However, it is possible to observe low percentages of Pd of zero state at the binding energies of 335.2 and 340.4 eV, which can be related to the effect of NaBH_4_ in the reduction reactions.

A hot filtration test was performed to confirm the effect of metal leaching on the catalytic activity of the catalyst over the reactions. For this purpose, the reduction reaction of nitrobenzene in the presence of Fe_3_O_4_@SiO_2_@polysalophen-Pd^(II)^ and NaBH_4_ was performed under optimal conditions and the catalyst was filtered after 30 minutes of reaction time. At this time, the %conversion was 46%, and then the reaction was allowed to proceed for another 30 minutes (corresponding to the reaction time for 11a to reach a yield of 96%). No detectable improvement was observed for product 11a, and after a further 30 minutes of reaction time, the % conversion was 46%. The results show the heterogeneous performance of the catalyst in the reaction and its high catalytic activity. Therefore, the results showed well that the amount of leaching shown in the previous section is not enough to cause the reduction reaction and again confirms that the amount of leaching of the metal is very small. In addition, according to the results of the control experiments, Pd salt alone has an efficiency of about 5% (based on GC), and even this small amount was not observed for the leaching test.

## Conclusion

Finally, a domino reduction/MCR protocol has been developed for the efficient and direct transformation of nitroarenes to the corresponding 1-substituted-1*H*-1,2,3,4-tetrazoles under mild conditions using a Pd(ii)-polysalophen complex as an efficient heterogeneous nanocatalyst. The polysalophen ligand with a possible network structure was prepared on a triazine framework *via* a polycondensation reaction. Furthermore, reduction of nitroarenes has been performed to the corresponding amines with high to excellent selectivity. Upon kinetics studies, the nitro reduction catalyzed by the Pd(ii)-polysalophen complex follows first-order kinetics, in agreement with the literature. Insignificant metal leaching, mild reaction conditions, an immobilized strong salophen ligand, high selectivity towards reduction, and stability during consecutive recycling without any deactivation were some of the advantages of the present method, which makes it a potential and suitable alternative method for reduction as well as MCR protocols compared to other reported methods.

## Data availability statement

The data that supports the findings of this study are available in the ESI† of this article.

## Conflicts of interest

There are no conflicts to declare.

## Supplementary Material

RA-011-D1RA01164B-s001

## References

[cit1] Goswami A., Kadam R. G., Tuček J., Sofer Z., Bouša D., Varma R. S., Gawande M. B., Zbořil R. (2020). Chem. Eng. J..

[cit2] Ansari S., Khorshidi A., Shariati S. (2020). RSC Adv..

[cit3] Dasgupta H. R., Mukherjee S., Ghosh P. (2019). Tetrahedron Lett..

[cit4] Gholinejad M., Esmailoghli H., Sansano J. M. (2020). Can. J. Chem..

[cit5] Nasrollahzadeh M., Nezafat Z., Gorab M. G., Sajjadi M. (2020). J. Mol. Catal..

[cit6] Kumar A., Paul B., Boukherroub R., Jain S. L. (2020). J. Hazard. Mater..

[cit7] Deshmukh D. G., Bangal M. N., Kalawade K. A., Mathad V. T. (2020). Org. Process Res. Dev..

[cit8] Zeynizadeh B., Aminzadeh F. M., Mousavi H. (2019). Res. Chem. Intermed..

[cit9] Mahmoudi B., Rostami A., Kazemnejadi M., Hamah-Ameen B. A. (2020). J. Mol. Catal..

[cit10] Mittal R., Awasthi S. K. (2019). Synthesis.

[cit11] Rostami-Vartooni A., Alizadeh M., Bagherzadeh M. (2015). Beilstein J. Nanotechnol..

[cit12] Neochoritis C. G., Zhao T., Dömling A. (2019). Chem. Rev..

[cit13] Aridoss G., Laali K. K. (2011). Eur. J. Org. Chem..

[cit14] Zhang F. G., Chen Z., Cheung C. W., Ma J. A. (2020). Chin. J. Chem..

[cit15] Varadaraji D., Suban S. S., Ramasamy V. R., Kubendiran K., Raguraman J. S. K., Nalilu S. K., Pati H. N. (2010). Org. Commun..

[cit16] Malik M. A., Al-Thabaiti S. A., Malik M. A. (2012). Int. J. Mol. Sci..

[cit17] May B. C., Abell A. D. (2002). J. Chem. Soc., Perkin Trans. 2.

[cit18] Nasseri M. A., Rezazadeh Z., Kazemnejadi M., Allahresani A. (2021). Catal. Lett..

[cit19] Strohmann M., Vossen J. T., Vorholt A. J., Leitner W. (2020). Green Chem..

[cit20] He Y., Wang X. (2020). Org. Lett..

[cit21] Li W., Zhang C., Lu H., Wang Y., Deng G., Liang Y., Yang Y. (2020). Org. Chem. Front..

[cit22] Hwu J. R., Roy A., Panja A., Huang W. C., Hu Y. C., Tan K. T., Lin C. C., Hwang K. C., Hsu M. H., Tsay S. C. (2020). J. Org. Chem..

[cit23] Ge S., Zhang Y., Tan Z., Li D., Dong S., Liu X., Feng X. (2020). Org. Lett..

[cit24] Cozzi P. G. (2004). Chem. Soc. Rev..

[cit25] Yao X., Chen H., Lü W., Pan G., Hu X., Zheng Z. (2000). Tetrahedron Lett..

[cit26] Song Y., Yao X., Chen H., Pan G., Hu X., Zheng Z. (2002). J. Chem. Soc., Perkin Trans. 2.

[cit27] Wang Y., Yang X., Yu J. (2017). RSC Adv..

[cit28] Wu S., Lu S. (2003). J. Mol. Catal. A: Chem..

[cit29] Solomon M. B., Rawal A., Hook J. M., Cohen S. M., Kubiak C. P., Jolliffe K. A., D'Alessandro D. M. (2018). RSC Adv..

[cit30] Cheng S., Wei W., Zhang X., Yu H., Huang M., Kazemnejadi M. (2020). Green Chem..

[cit31] Kazemnejadi M., Shakeri A., Nikookar M., Mohammadi M., Esmaeilpour M. (2017). Res. Chem. Intermed..

[cit32] Thomas J. M., Raja R., Sankar G., Bell R. G. (2001). Acc. Chem. Res..

[cit33] Kazemnejadi M., Alavi G S. A., Rezazadeh Z., Nasseri M. A., Allahresani A., Esmaeilpour M. (2019). Green Chem..

[cit34] Kazemnejadi M., Alavi G S. A., Rezazadeh Z., Nasseri M. A., Allahresani A., Esmaeilpour M. (2020). Appl. Organomet. Chem..

[cit35] Kazemnejadi M., Ahmed R. O., Mahmoudi B. (2020). RSC Adv..

[cit36] Sardarian A. R., Kazemnejadi M., Esmaeilpour M. (2019). Dalton Trans..

[cit37] Fuertes A. B., Valle-Vigón P., Sevilla M. (2012). Chem. Commun..

[cit38] Yang T., Liu J., Zheng Y., Monteiro M. J., Qiao S. Z. (2013). Chem. - Eur. J..

[cit39] Liu R., Qu F., Guo Y., Yao N., Priestley R. D. (2014). Chem. Commun..

[cit40] Sun C., Sun K., Tang S. (2018). Mater. Chem. Phys..

[cit41] Nasseri M. A., Rezazadeh Z., Kazemnejadi M., Allahresani A. (2020). Dalton Trans..

[cit42] Habibi D., Pakravan N., Arabi A., Kaboudvand Z. (2018). Appl. Organomet. Chem..

[cit43] Kazemnejadi M., Mahmoudi B., Sharafi Z., Nasseri M. A., Allahresani A., Esmaeilpour M. (2020). Appl. Organomet. Chem..

[cit44] Nasseri M. A., Alavi S. A., Kazemnejadi M., Allahresani A. (2019). RSC Adv..

[cit45] Devi T. B., Ahmaruzzaman M., Begum S. (2016). New J. Chem..

[cit46] Gu S., Wunder S., Lu Y., Ballauff M., Fenger R., Rademann K., Jaquet B., Zaccone A. (2014). J. Phys. Chem..

[cit47] Nanaei M., Nasseri M. A., Allahresani A., Kazemnejadi M. (2019). SN Appl. Sci..

[cit48] Aditya T., Pal A., Pal T. (2015). Chem. Commun..

[cit49] Herves P., Perez-Lorenzo M., Liz-Marzan L. M., Dzubiella J., Lu Y., Ballauff M. (2012). Chem. Soc. Rev..

[cit50] Aitenneite H., Abboud Y., Tanane O., Solhy A., Sebti S., Bouari A. E. (2016). J. Mater. Environ. Sci..

[cit51] Li Z., Xu X., Jiang X., Li Y., Yu Z., Zhang X. (2015). RSC Adv..

[cit52] Baba A., Ouahbi H., Hassine A., Sebti J., Laasri L., Sebti S. (2018). Mediterr. J. Chem..

[cit53] Vivek S., Arunkumar P., Babu K. S. (2016). RSC Adv..

[cit54] Chekalil N., Tarhini M., Elaissari A., Saïdi-Besbes S. (2019). Res. Chem. Intermed..

[cit55] Masoule S. F., Pourhajibagher M., Safari J., Khoobi M. (2019). Res. Chem. Intermed..

