# Supplementary material for: Efficient reduction of nitro compounds and domino preparation of 1-substituted-1H-1,2,3,4-tetrazoles by Pd(ii)-polysalophen coated magnetite NPs as a robust versatile nanocomposite
Source: RSC Adv. 2021 Mar 30;11(21):12484–99. doi: 10.1039/d1ra01164b (PMC8697099; doi:10.1039/d1ra01164b)
Supplement: RA-011-D1RA01164B-s001 [file RA-011-D1RA01164B-s001.pdf]

Electronic Supplementary Material (ESI) for RSC Advances.
This journal is © The Royal Society of Chemistry 2021

Supplementary materials

Efficient reduction of nitro compounds and domino preparation of 1-substituted-1*H*-1,2,3,4-tetrazoles by Pd(II)-polysalophen coated magnetite NPs as a robust versatile nanocomposite

DaPeng Xu,[a] Meilu Xiong,[b]*Milad Kazemnejadi,*[c]

[a] *Institute of Chemical Technology, Gansu Industry Polytechnic College, TianShui 741000, China Public Foundation College, Gansu Health Vocational College, Lanzhou 730207 , China*
[b] *Department of Chemistry, Faculty of Sciences, University of Birjand, Birjand, Iran.*
**Corresponding: Email: bearbabyx@sina.com (Meilu Xiong); miladkazemnejad@yahoo.com (Milad Kazemnejadi)*

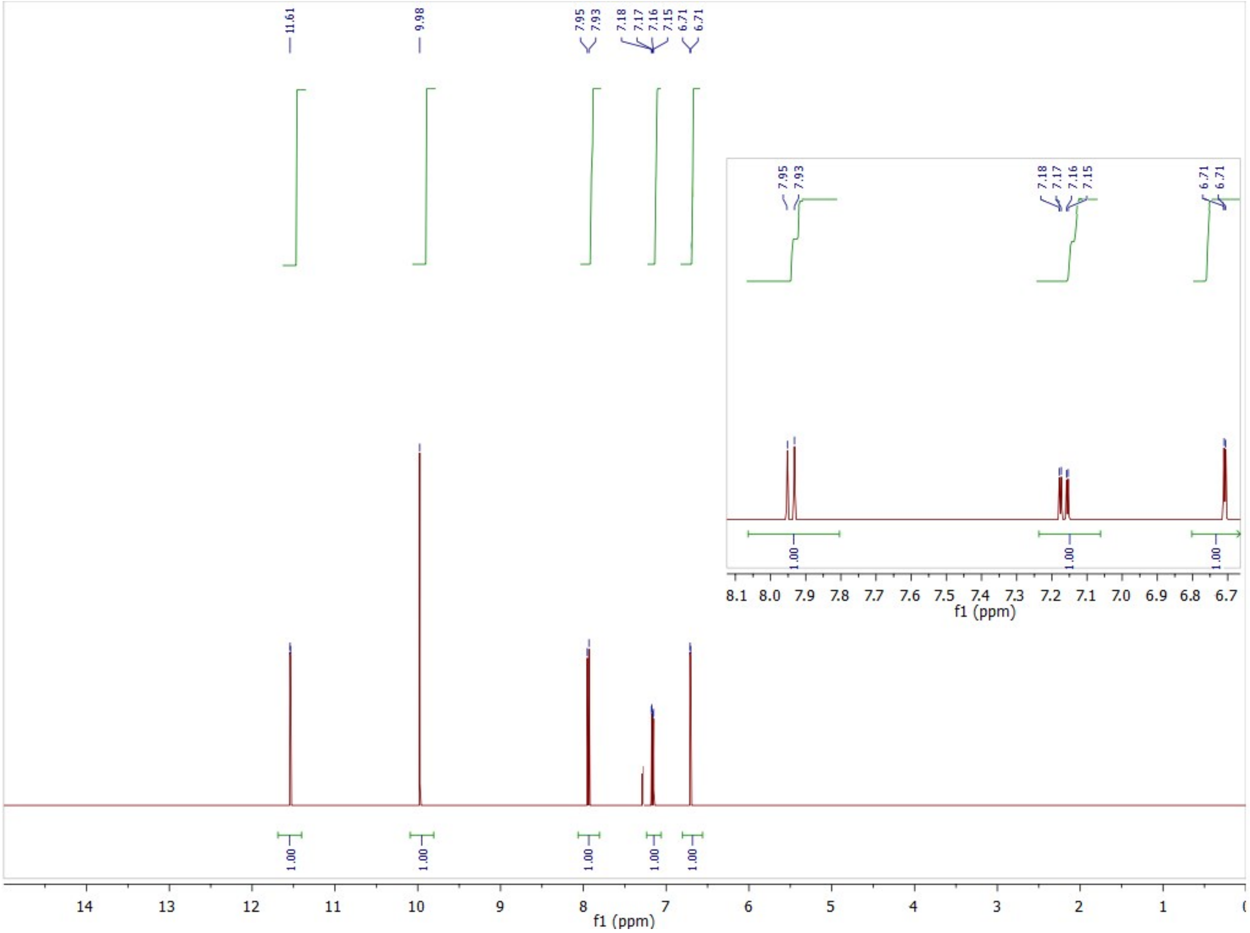

Fig. S1 ^{1}H-NMR spectrum of TCT(SA)$_3$ (**3**) ($CDCl_3$, 400 MHz)

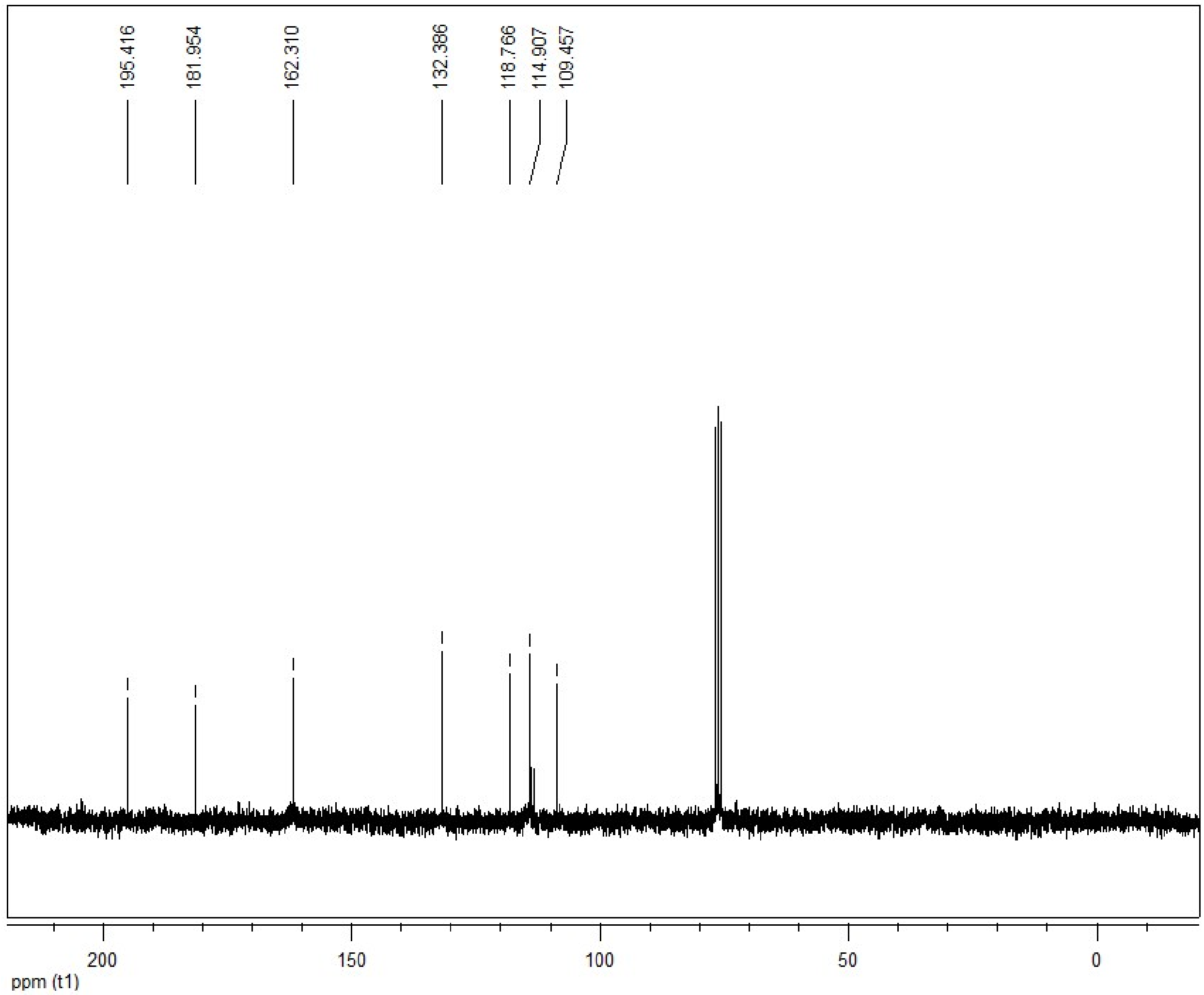

Fig. S2 ^{13}C-NMR spectrum of TCT(SA)$_3$ (**3**) (CDCl$_3$, 100 MHz)

Table S1 Determination of Pd content and mean diameter in Cat. **9** with different Pd loading amounts

Entry	Cat. **9**/ Pd (wt%)		Mean diameter (nm)[a]
	ICP analysis	EDX (mean of 5 points)	
1	2.22	1.54	82
2	6.06	5.90	85
3	10.11	9.90	82
4	14.16	13.98	90
5	18.19	18.01	94

[a] DLS analysis

Table S2 Reduction of nitroarens and domino preparation of 1-substituted-1*H*-1,2,3,4-tetrazoles catalyzed by Fe_3O_4@SiO_2@polysalophen-Pd(II) **9** with various Pd content.

Entry	Catalyst **9**	Product		
		**7a**[a]	**8a**[b]	**8a**[c]

		Time (min)	Yield (%)	Time (min)	Yield (%)	Time (min)	Yield (%)
1	Cat. **9**/ 0.12 mol%Pd	60	33	75	22	90	15
2	Cat. **9**/ 0.3 mol%Pd	60	72	75	60	90	50
3	Cat. **9**/ 0.6 mol%Pd	60	96	75	97	90	98
4	Cat. **9**/ 0.8 mol%Pd	60	98	75	97	90	98
5	Cat. **9**/ 1.0 mol%Pd	60	95	75	92	90	90

[a] Nitroarene (1.0 mmol), $NaBH_4$ (2.0 mmol), H_2O (2.0 mL), catalyst **9** (5 mg), reflux.

[b] Aniline (1.0 mmol), triethyl orthoformate (1.2 mmol), catalyst **9** (5 mg), H_2O (2.0 mL), reflux.

[c] Nitrobenzene (1.0 mmol), triethyl orthoformate (1.2 mmol), NaN_3 (1.5 mmol), $NaBH_4$ (2.0 mmol), H_2O (2.0 mL), catalyst **9** (5 mg), reflux.

Characterization data for 12a-12h

1-Phenyl-1*H*-Tetrazole (Table 3, 12a)

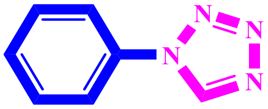

White solid; m.p. 64-45 °C [1]; ^{1}H NMR (250 MHz, $CDCl_3$) δ(ppm)= 6.87-7.05 (m, 5H), 8.20 (s, 1H); ^{13}C NMR (62.9 MHz, $CDCl_3$) δ(ppm)= 129.5, 130.1, 134.2, 135.0; CHN: Found %: C 57.52; H 4.10; N 38.39. $C_7H_6N_4$. Calculated, %: C 57.53; H 4.14; N 38.34.

1-(4-Methoxyphenyl)-1*H*-Tetrazole (Table 3, 12b)

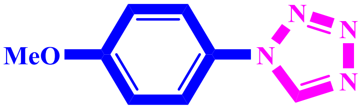

White solid; m.p. 114-115 °C [1]; ^{1}H NMR (250 MHz, $CDCl_3$) δ(ppm)= 3.72 (s, 3H), 6.72 (d, *J*= 8.00 Hz, 2H), 7.19 (d, *J*= 8.00 Hz, 2H), 8.27 (s, 1H); ^{13}C NMR (62.9 MHz, $CDCl_3$) δ(ppm)= 57.8, 115.4, 123.6, 128.4, 133.6, 150.6; CHN: Found %: C 54.52; H 4.60; N 31.81. $C_8H_8N_4O$. Calculated, %: C 54.54; H 4.58; N 31.80.

1-(4-Phenol)-1*H*-Tetrazole (Table 3, 12c)

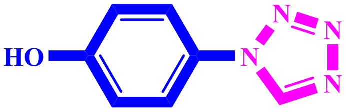

White solid; mp 185-187 °C [1,2]; ^{1}H NMR (250 MHz, DMSO-d_6) δ(ppm)= 6.97-7.00 (d, *J*= 9 Hz, 2 H), 7.6-7.7 (d, *J*= 9 Hz, 2 H), 9.94 (s, 1 H), 10.15 (s, 1H); ^{13}C NMR (62.9 MHz, DMSO-d_6) δ(ppm)= 116.6, 123.5, 125.9, 142.5, 159.0; CHN: Found %: C 51.87; H 3.76; N 34.50. $C_7H_6N_4O$. Calculated, %: C 51.85; H 3.73; N 34.55.

1-*p*-Tolyl-1*H*-Tetrazole (Table 3, 12d)

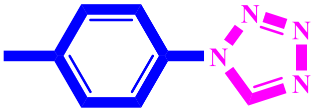

White solid (91% yield); m.p. 94-96 °C [1,3]; ^{1}H NMR (250 MHz, $CDCl_3$) δ(ppm)= 2.87 (s, 3H), 6.73 (d, *J*= 8.50 Hz, 2H), 7.15 (d, *J*= 8.50 Hz, 2H), 8.27 (s, 1H); ^{13}C NMR (62.9 MHz, $CDCl_3$) δ(ppm)= 23.8, 116.7, 124.9, 127.5, 133.6, 145.4; CHN: Found %: C 59.93; H 3.5.05; N 35.02. $C_8H_8N_4$. Calculated, %: C 59.99; H 5.03; N 34.98.

1-(4-Chlorophenyl)-1*H*-Tetrazole (Table 3, 12e)

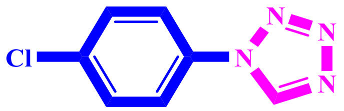

White crystal; m.p. 157 °C [3,4]; ^{1}H NMR (250 MHz, $CDCl_3$) δ(ppm)= 7.30 (d, J= 9.0 Hz, 2H), 7.51 (d, J= 9.6 Hz, 2H), 8.36 (s, 1H); ^{13}C NMR (62.9 MHz, $CDCl_3$) δ(ppm)= 117.7, 119.1, 123.7, 129.5, 135.8; CHN: Found %: C 46.55; H 2.77; N 31.06. $C_7H_5ClN_4$. Calculated, %: C 46.56; H 2.79; N 31.02.

1-(4-Benzonitrile)-1*H*-Tetrazole (Table 3, 12f)

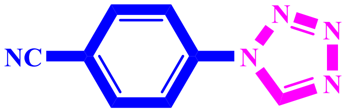

White solid; m.p. 177-179°C [1,2]; ^{1}H NMR (250 MHz, $CDCl_3$) δ(ppm)= 7.25 (d, J= 5.75 Hz, 2H), 7.32 (d, J= 5.7 Hz, 2H), 8.26 (s, 1H); ^{13}C NMR (62.9 MHz, $CDCl_3$) δ(ppm)= 112.3, 118.2, 122.7, 132.8, 137.4, 140.4; CHN: Found %: C 56.17; H 2.97; N 40.86. $C_8H_5N_5$. Calculated, %: C 56.14; H 2.94; N 40.92.

2-(1*H*-tetrazol-1-yl) pyridine (Table 3, 12g)

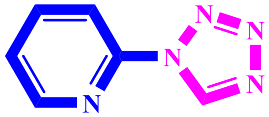

White solid; mp 80-82 °C [18]; ^{1}H NMR (250 MHz, $CDCl_3$) δ(ppm)= 7.50-7.45 (m, 1 H), 8.05-7.99 (m, 1 H), 8.12-8.09 (d, J= 9 Hz, 1 H), 8.57-8.55 (q, J= 2.3Hz, 1 H), 9.57 (s, 1 H); ^{13}C NMR (62.9 MHz, $CDCl_3$) δ(ppm)= 114.4, 124.8, 139.8, 140.1, 146.9, 149.0; CHN: Found %: C 49.01; H 2.45; N 46.55. $C_6H_5N_5$. Calculated, %: C 48.98; H 3.43; N 47.60.

1-(3-Nitrobenzene)-1*H*-Tetrazole (Table 3, 12h)

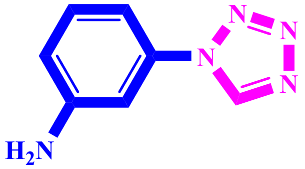

White solid; m.p. 106-108 °C [2,4]; ^{1}H NMR (250 MHz, $CDCl_3$) δ(ppm)= 7.41-7.48 (m, 1 H), 7.90 (d, 1 H, J = 9.5 Hz), 8.02 (d, 1 H, J = 9.5 Hz), 8.40 (s, 1 H), 8.53 (s, 1 H); ^{13}C NMR (62.9 MHz, $CDCl_3$) δ(ppm)= 114.4, 118.6, 125.5, 129.7, 130.4, 139.1, 149.8; CHN: Found %: C 52.19; H 4.41; N 43.40. $C_7H_7N_5$. Calculated, %: C 52.17; H 4.38; N 43.45.

References

[1] Habibi, D., Pakravan, N., Arabi, A., & Kaboudvand, Z. (2018). Preparation of Fe_3O_4@ 5, 10-dihydropyrido [2, 3-b] quinoxaline-7, 8-diol copper complex: A capable nanocatalyst for the green synthesis of 1-substituted 1*H*-tetrazoles. *Applied Organometallic Chemistry*, 32(1), e3988.

[2] Aridoss, G., & Laali, K. K. (2011). Building Heterocyclic Systems with $RC(OR)^{2+}$ Carbocations in Recyclable Brønsted Acidic Ionic Liquids: Facile Synthesis of 1-Substituted

1*H*-1, 2, 3, 4-Tetrazoles, Benzazoles and Other Ring Systems with $CH(OEt)_3$ and $EtC(OEt)_3$ in $[EtNH_3][NO_3]$ and $[PMIM (SO_3H)][OTf]$. *European Journal of Organic Chemistry*, *2011*(15), 2827-2835.
[3] Sharghi, H., Ebrahimpourmoghaddam, S., & Doroodmand, M. M. (2013). Facile synthesis of 5-substituted-1H-tetrazoles and 1-substituted-1H-tetrazoles catalyzed by recyclable 4′-phenyl-2, 2′: 6′, 2 ″-terpyridine copper (II) complex immobilized onto activated multi-walled carbon nanotubes. *Journal of Organometallic Chemistry*, *738*, 41-48.
[4] Ghasemzadeh, M. S., & Akhlaghinia, B. (2017). 2-Aminoethanesulfonic Acid Immobilized on Epichlorohydrin Functionalized Fe_3O_4@WO_3 (Fe_3O_4@WO_3-EAE-SO_3H): A Novel Magnetically Recyclable Heterogeneous Nanocatalyst for the Green One-Pot Synthesis of 1-Substituted-1 *H*-1, 2, 3, 4-Tetrazoles in Water. *Bulletin of the Chemical Society of Japan*, *90*(10), 1119-1128.
